# Multifunctional material platforms for neural interfaces: active orchestration of dynamic foreign body response across implantation lifetimes

**DOI:** 10.1016/j.bioactmat.2026.06.037

**Published:** 2026-06-28

**Authors:** Wei Yao, Yueyue Li, Yuanyuan Meng, Xiaoling Pan, Zhenyu Sun, Yuan Gao, Xiaodong Zhu

**Affiliations:** a Center for Translational Medicine Research, Naval Medical University, Shanghai, 200433, PR China; b Department of Traditional Chinese Medicine, Changzheng Hospital, Naval Medical University, Shanghai, 200433, PR China; c Key Loboratory of Resource Biology and Biotechnology in Western China, Ministry of Education, College of Life Science, Northwest University, Xi'an, 710069, PR China

**Keywords:** Neural electrodes, Foreign body response, Immunomodulation, Long-term stability, Biomimetic interfaces

## Abstract

The sustained reliability of invasive brain-computer interface (BCI) electrodes is fundamentally constrained by progressive interface destabilization, a process driven by the dynamic foreign body response (FBR). Given the intricate, time-dependent evolution of the FBR, the establishment of long-term stable neural interfaces necessitates the deployment of sophisticated material architectures capable of intercepting core regulatory mechanisms across distinct pathological phases. This review synthesizes bio-inspired and functional material design strategies, systematically examining their capacity to actively modulate the FBR in a stage-specific manner. Specifically, these approaches are engineered to attenuate acute inflammatory cascades, which is hypothesized to impede detrimental glial scarring—while establishing robust biological barriers resilient to chronic biofouling and infection. Furthermore, by mitigating material degradation and micromotion-induced fretting, these strategies are associated with preserved the functional integrity of the interface over extended periods. By consolidating the theoretical principles, recent advancements, and persisting challenges associated with these material paradigms, this work aims to delineate a forward-looking framework for the development of ultra-durable BCI electrodes, thereby accelerating the clinical translation of neural interface technologies.

## Highlights

• Propose a new paradigm of “time-phase specificity design” for neural interfaces.
• Regulate acute inflammation and chronic biofouling through biomimetic materials.
• Utilize self-healing and flexible structures to achieve long-term stability.
• Addressing engineering conflicts and challenges in multifunctional coupling.
• Provide a forward-looking framework for the development of ultra-durable brain-computer interfaces.

## Graphical abstract

**Figure:**
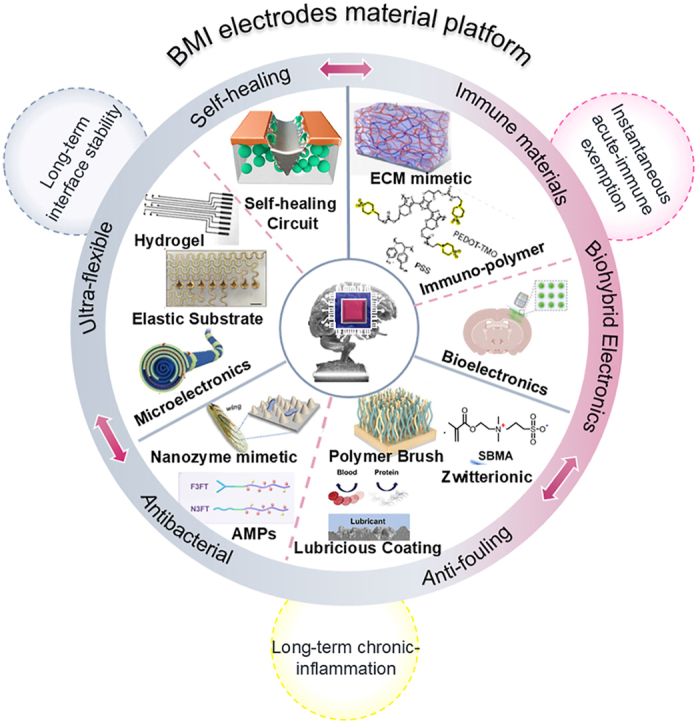

## 1 Introduction

**Fig. 1:**
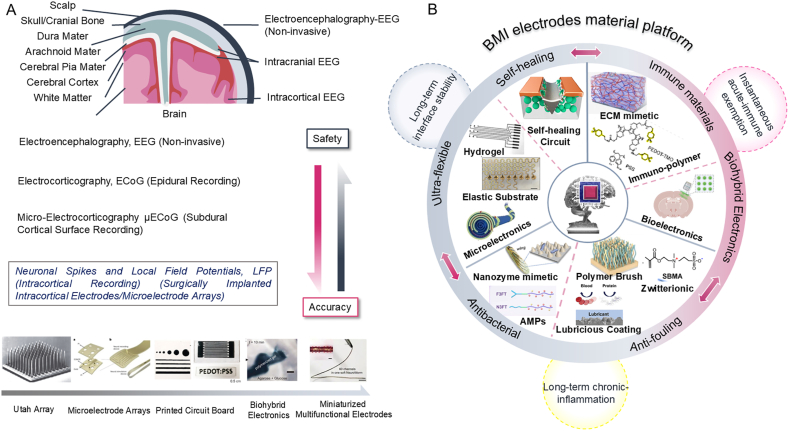
(A) Basic classification display of brain structure, signals, and interface electrodes; (B) Design principles and strategies for highly biocompatible brain-computer interface electrode material platforms.

The comprehensive interrogation of neural circuitry not only illuminates the complexities of human cognition and behavior but also catalyzes paradigm shifts in neuromodulation science. Such progress is pivotal for the diagnosis and therapeutic intervention of major neurological and neurodegenerative disorders, ranging from Alzheimer's and Parkinson's disease to psychiatric conditions and spinal cord injuries [1,2]. In this context, brain-computer interfaces (BCIs)—functioning as direct conduits between neural tissues and external artificial systems—hold substantial promise in restoring motor function for patients with impairments [3], enabling functional substitution, and augmenting cognitive capabilities [4]. Furthermore, these interfaces afford an innovative avenue for deciphering brain function and developing targeted therapeutic strategies [5]. Classified by invasiveness, BCIs are generally categorized into non-implantable [6,7], semi-implantable, and implantable modalities [8]. The intrinsic link between deep brain architectures (e.g., the basal ganglia and thalamus) and core physiological processes—spanning motor control, emotional regulation, and circadian rhythms—renders these regions primary targets for invasive neuromodulation techniques, such as deep brain stimulation (DBS) (Fig. 1A). Notably, implantable BCIs exhibit superior capacity in capturing neural signals with high spatiotemporal resolution, ranging from electrocorticography (ECoG) and multi-unit activity (MUA) to local field potentials (LFP) and single-neuron spikes [9]. By providing access to the most fundamental neural coding data, these devices ensure the high-fidelity transmission of stable electrophysiological signals [10]. This bidirectional capability facilitates both the decoding of intrinsic signals for external device control and the delivery of electrical stimuli to modulate target neurons, thereby permitting the assessment of neural correlates associated with gait recovery (see Table 1, Table 2).

**Table 1:** Core performance parameters of flexible electrodes.

Material/Device Name	Key Constituents	Fabrication Method/Structure	Young's Modulus	Mechanical Deformation	Electrical Properties	Primary Application(s)	Ref.
TRAIN	CNTs, PVA	Bottom-up approach	N/A	Tensile Strain: 64.5 ± 7.9%	Impedance: 33.20 ± 9.27 kΩ	Intraspinal Neural Recordings (in vivo)	[11]
HybF	PLGA, Alginate-MXene, Alginate-Ca^2+^, PEGDA	Wet-spinning	<200 kPa	Bending Stiffness: 0.3 N/m	Conductivity: 97 S/m	Suppression of Epileptic Seizures	[12]
OECT	PEDOT:PSS, PAM, PIL	Double-Network Semiconducting Polymer Gel	N/A	Tensile Strain: 50%	Transconductance: 86.4 mS	Stretchable e-skins, Artificial Synapses, Gas Sensors	[13]
hydro-SCs	g2T-T, AAc	Solvent Affinity-Induced Assembly	81 kPa	Tensile Strain: 150%	Charge Carrier Mobility: 1.4 cm^2^ V^−1^ s^−1^	Biointerfacing, Biochemical Sensing	[14]
PDEH-SLM	EGaIn, Ag Flakes, PAA, PASP-DA	Multilayer Patches	3.4 kPa	Tensile Strain: 1760%	Conductivity: 1329.7 S cm^−1^	Diagnosis of Peripheral Neuropathy	[15]
BOIEFI	PI, Ecoflex	UV Laser-Cutting; Root-like Structures	N/A	Elongation at Break: 540%	N/A	Soft Pressure Sensors	[16]
SMNEA	PMMA, PI, Cr/Au, Ti/SiO_2_	Laser Micromachining; Transfer Printing	6.6 GPa	Stretchability: 60-90%	N/A	Bioelectronic Interfaces	[17]
SMBE	PI, PEDOT:PSS, Cr/Au	2D Fabrication; 3D Structuring	N/A	Compressive Strain (Cyclic): 50-100%	Charge Storage Capacity: 27.98 mC cm^−2^	Minimally Invasive Brain-Computer Interface	[18]
LM@SF-PAA	Silk Fibroin, Liquid Metal	Core-Shell Particle Coordination	22.2 kPa	Tensile Strain: 1050%	Conductivity: 0.245 S m^−1^	Portable Flexible ECG Monitoring	[19]
PTPL	PVP, Liquid Metal Particles	Self-Assembling	175.86 kPa	Tensile Strain: >20,000%	Sheet Resistance: 26.19 mΩ sq^−1^	Epidermal Electrode	[20]
Bio-patch	CS, PVP, PLLA, PLGA	Sequential Electrospinning	9.73 MPa	Tensile Strain: 187%	N/A	Liver Repair Orchestration; Renal Injury Regeneration	[21]
CNT Array	CNT, Al_2_O_3_	VACNT Forest; Densification & Polymer Infiltration	∼54 MPa	N/A	Impedance Density: ∼0.015 kΩ μm^−2^	Biocompatible Neural Recording	[22]
Interfacial PN	SIBS, AgNWs, PP-g-MAH	Hybrid 2D/3D Percolation Network	N/A	Elongation at Break: >660%	Conductivity: 46,750 S cm^−1^	Biomechanical Sensing	[23]
SIB	AgNW, PUA	Layered Architectural Composite	∼10 MPa	Elongation at Break: >150%	Sheet Resistance: ∼1 Ω sq^−1^	Sciatic Nerve Stimulation	[24]
rGPF	rGO, PANI	Self-Assembly	3.73 ± 0.47 GPa	Bending Stiffness (at 800 nm wavelength): Suitable	N/A	Investigation of Brain Events & Disease Mechanisms	[25]
NeuroWorm	SEBS, PET	2D-to-1D Rolling Transformation	3.1 ± 0.2 MPa	Elongation at Break: 93 ± 3.2%	Resistance: 20.6 GΩ	Electrophysiological & Mechanical Signal Detection	[26]
I3K/SF-SF	Silk Fibroin, I3K Peptide	"W"-shaped Structural	26.4 kPa	N/A	N/A	Biodegradable Piezoelectric Sensors (in vivo)	[27]

**Table 2:** Main text sections – Evidence level cross-reference table.

Manuscript Section	Subsection	Grade 2 (highly relevant): peripheral nerve implantation	Grade 3 (analogous implantation): non-neural implants/wound repair/bone repair	Grade 4 (mechanistic inspiration): flexible electronics	Grade 5 (conceptual background): materials science
Section 2	2.2 Bionic ECM	58	54,55	N/A	56, 63, 64, 66-72
	2.3 Immune Functional Polymer	76,81	79,80	N/A	N/A
	2.4 Biohybrid electronics	N/A	N/A	N/A	N/A
Section 3	3.1 Antifouling surface design	107,114	99, 101, 104, 109,111,112,116,127,128,130,133,146,91, 98, 115, 124,141,144,147	94, 100, 106, 108	92, 93, 97, 103,110,121,123,125,126,129,131,132,134,135,142,143,148
	3.2 Antibacterial coating	166,189	162,171,164, 167, 170, 172-174,176,178,180,193	163,165	169, 177, 192
Section 4	4.1 Adaptive super flexibility	201, 204, 206,208, 225	215,218,220,221,228,240,203, 211	205,210,212–214,216,217,222,224,232,235-238	209
	4.2 Self-healing performance	243, 246, 263	241, 251, 252,257, 264	242, 256, 259, 266-268,272,277	253, 260, 261,265, 271,279
Section 5	Engineering implementation and clinical translation barriers	N/A	N/A	N/A	N/A

Generally, a BCI system encompasses signal acquisition, information decoding, and control output modules. As the critical physical interface, implantable electrodes [8]. necessitate low interfacial impedance, high charge-injection capacity, robust chronic stability, and biocompatibility [28]. However, the long-term utility of these devices is fundamentally constrained by the foreign body response (FBR), a progressive biological reaction that threatens interface stability [[29], [30], [31]]. The primary impediment arises from implantation-induced neuroinflammation, which not only compromises neuronal viability but also precipitates the formation of dense glial scarring. This process creates a physical isolation barrier, continuously degrading the local microenvironment and underscoring the limitations of current biocompatibility designs in fostering beneficial tissue integration [10,32]. Compounding this challenge is the chronic accumulation of biofouling—characterized by protein adsorption, extracellular matrix deposition, and tissue hyperplasia—which correlates with a substantial elevation in electrode-tissue interface impedance. This degradation trajectory is associated with impaired the signal-to-noise ratio and spatial resolution while diminishing the efficiency of neural stimulation, ultimately resulting in a systematic decline in signal fidelity. Furthermore, the mechanical mismatch between the rigid implant and the dynamic brain tissue—subject to respiration, blood flow, and micromotion—combined with a persistent inflammatory milieu, exerts continuous stress and chemical corrosion on the device. This dual mechanism significantly heightens the risk of electrode displacement, structural failure, or encapsulation, highlighting the formidable technical hurdles in guaranteeing ultra-long-term structural integrity within a dynamic physiological environment.

To circumvent these multifaceted challenges, this review delineates a “temporal stage-specific design” paradigm (Fig. 1B). Herein, this paradigm is defined as a full-lifecycle, FBR evolution-driven material design framework for neural interfaces. Based on the sequential cascade of FBR across the entire implantation cycle—acute inflammatory initiation, chronic biofouling/infection progression, and long-term structural failure—this framework deploys targeted material strategies that precisely match the dominant pathological mechanism of each stage, and realizes synergistic, non-conflicting regulation of the entire implantation cycle through systematic integration of stage-specific strategies. This framework is distinct from two conventional research paradigms: first, it differs from traditional staged intervention strategies that focus only on acute inflammation suppression, as it covers the full FBR cascade from implantation to long-term failure; second, it avoids the functional conflicts common in conventional multifunctional designs that simply superimpose unrelated functions, by aligning material functionalization with the temporal progression of pathology.

Predicated on the dynamic evolution of the FBR, this framework addresses failure mechanisms intrinsic to specific pathological phases: for the acute phase dominated by inflammation, the strategy prioritizes immunomodulation to inhibit scarring and guide microenvironmental repair; for chronic stages prone to biofouling and infection, the focus shifts to constructing biological barriers via antifouling and antibacterial integration; regarding structural instability driven by corrosion and micromotion, the design incorporates self-healing and flexible materials to ensure longevity. Rather than operating in isolation, these phased strategies are systematically integrated through bio-inspired and functional design principles. This review aims to construct a feedback-sensitive temporal framework, clarifying its theoretical underpinnings and implementation pathways, thereby laying a foundation for next-generation BCI platforms capable of achieving stable, efficient, and long-term brain-machine integration. In this review, we clearly distinguish the material strategies directly validated in in vivo intracranial neural electrode models from the prospective translational references derived from adjacent implantable biomaterial fields, to clarify the evidence level and application boundary of each design paradigm.

## 2 Mitigate acute-phase immune responses

### 2.1 The process of immune response

**Fig. 2:**
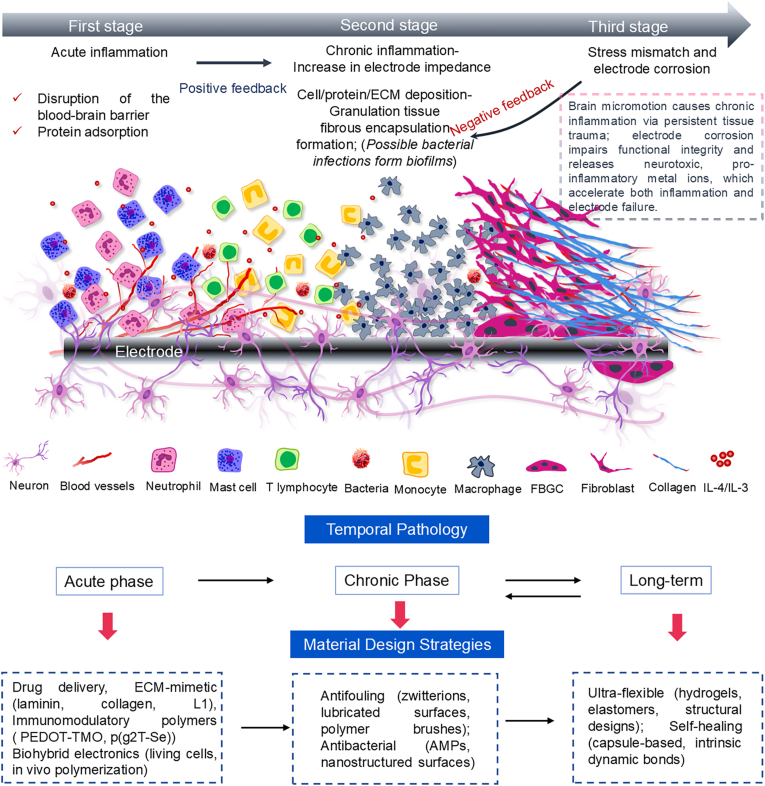
Temporal progression of pathological events after electrode implantation and the corresponding material design strategies.

This chapter details the material strategies for the acute inflammatory phase, which is the initiating regulatory link of the temporal stage-specific design paradigm. The implantation of neural electrodes into brain tissue inevitably triggers a foreign body reaction (FBR), a complex biological response common to all implanted materials. As illustrated in Fig. 2, the time-dependent foreign body reaction during the implantation process is summarized. This is because the implantation of neural electrodes is inherently an invasive procedure; even surgeries that avoid the major arteries and veins of the pia mater inevitably lead to the rupture of cortical capillaries. The first and most critical event occurring upon electrode implantation is the disruption of the blood-brain barrier (BBB) [33]. This process initiates as the electrode advances toward the target site: its mechanical displacement inevitably lacerates and abrades the traversing brain tissue, disrupting the normal architecture of blood vessels, cells, and the extracellular matrix (ECM). Consequently, blood and interstitial fluid come into contact with the microelectrode surface. Blood cells and plasma proteins released from ruptured vessels rapidly adsorb onto the microelectrode surface within seconds to minutes, forming a complex, transient protein and cellular matrix layer accompanied by inflammatory cell infiltration [34,35]. Furthermore, neuroinflammation is associated with enhanced BBB permeability [36], facilitating the extravasation of blood-borne components, such as fibrinogen and thereby exacerbating brain tissue damage.

The influx of plasma proteins triggers inflammatory responses and immune cell recruitment [37]. Neuroinflammation is a complex process mediated by crosstalk among blood cells [38], endothelial cells [39], and glial cells (particularly microglia). Implantable electronic devices typically induce chronic inflammatory responses with sustained activation of astrocytes and microglia [40]. Within minutes following blood-brain barrier (BBB) disruption, microglia—the primary innate immune defenders of the nervous system—are rapidly activated and proliferate at the implantation site [[41], [42], [43]]. Their filopodia enable efficient participation in inflammatory cascades, the formation of foreign body giant cells (FBGCs), and phagocytosis of xenobiotic materials [44]. Subsequently, within hours post-implantation, astrocytes are activated by mediators including IL-1β, TNF-α, and complement component 1q (C1q) secreted by microglia, with a directional extension of cellular processes toward the injury locus [45]. Reactive astrocytes coordinate with microglia via signaling pathways involving IFN-γ and/or TNF-α cytokines and chemokines [46]. Within days of injury, NG2 glial cells undergo robust proliferation [47]. Notably, microglia initiate migration toward the microelectrode within 12–24 h, followed sequentially by NG2 glial cells. Astrocytic reactivity to central nervous system (CNS) injury exhibits pronounced heterogeneity; unlike microglia and NG2 glia, astrocytes do not migrate but instead undergo reactive alterations such as hypertrophy and hyperplasia. De novo astrocyte formation is detectable as early as 24 h post-implantation. Weeks later, these glial populations is associated with the formation of a dense glial scar that encapsulates the implanted probe, physically isolating the device from adjacent neuronal tissues and thereby correlating with impaired the efficient transduction of electrical signals [45]. In the long term, early-stage protein adsorption onto the electrode surface is associated with a regulatory platform that correlates with subsequent inflammatory responses and the recruitment, activation, and polarization of immune cells [48,49]. Thus, the precise modulation of early immune responses is hypothesized to be key for abrogating the excessive activation and progression of the immune cascade [50], and constitutes the fundamental prerequisite for achieving long-term interfacial stability of neural electrodes. Based on the temporal pathology described above, Fig. 2 (lower panel) presents an overview of the stage-specific material strategies. Sections 2, 2.1, 2.2 will critically compare these strategies with respect to their mechanisms, limitations, and suitability for different neural electrode systems.

Clinically, systemic administration regimens represent the mainstay for suppressing early-stage infections and mitigating inflammatory responses, encompassing the prophylactic use of antibiotics preoperatively and the sustained delivery of antibiotics combined with immunosuppressive agents postoperatively [51]. However, systemic drug delivery is plagued by inherent limitations, including poor targeting specificity, inadequate local effective concentrations, and potential systemic toxicities, which preclude achieving long-term, robust immune modulation. To overcome the limitations of systemic administration—poor targeting specificity and systemic toxicity—the direct immobilization of anti-inflammatory molecules onto the electrode surface has emerged as a conventional strategy. Among the diverse spectrum of anti-inflammatory agents employed clinically, dexamethasone (Dex)—a potent corticosteroid—has been extensively utilized for the management of various inflammatory disorders [52,53]. For instance, the immobilization of Dex within poly(3,4-ethylenedioxythiophene) (PEDOT) coatings yielded attenuated inflammatory responses and improved tissue integration following implantation into the hippocampal region of rats [54]. Building on this paradigm, researchers further developed a therapeutic cortical electrode by co-immobilizing tetracycline (TC) and Dex within bacterial cellulose (BC) hydrogels, which were subsequently integrated as flexible electrode substrates. This engineered electrode enabled stable recording of electrocorticogram (ECoG) signals in rat models, while simultaneously exerting potent efficacy in mitigating bacterial infections and inflammatory reactions induced by electrode implantation [55]. In recent years, the research focus has shifted toward a source-control strategy centered on material design and device fabrication, with the aim of developing neural electrodes endowed with intrinsic low immunogenicity that obviate the need for exogenous drug delivery. The suppression of immune activation via the inherent physicochemical properties of the electrodes themselves constitutes a fundamental solution to the progressive functional deterioration of devices, which is often triggered by suboptimal management of acute-phase immune responses during initial implantation.

Notably, the pathological cascade from foreign body reaction to glial scar formation and subsequent electrode performance decay represents a well-validated consensus mechanism supported by extensive in vivo histological and electrophysiological evidence. In contrast, the associations between material-mediated anti-inflammatory, anti-fibrotic, or anti-fouling properties and enhanced recording stability are primarily phenotypic correlations interpreted via mechanistic deduction, rather than definitive causal relationships validated by targeted experiments.

Focusing on the core pathological cascade of acute FBR after neural electrode implantation, the existing regulatory strategies are categorized into three major technical systems. These systems approach acute-phase inflammation regulation from fundamentally different underlying logics, exhibiting essential differences in action targets, intervention timelines, and functional boundaries. The first is biomimetic ECM materials, centered around physiological homogenization. Simulating the biochemical and mechanical properties of the extracellular matrix fundamentally reduces the foreign-body properties of materials, balancing anti-inflammatory effects and neural interface integration; The second is immune-functional polymers, centered around pathway-targeted intervention. Through molecular structure design, it regulates inflammatory signals and clears inflammatory mediators. It can be in-situ compounded with the conductive layer of electrodes without altering the electrodes' original structure; The last is biohybrid electronics, centered on eliminating foreign-body properties. Through live-cell preloading and in-situ biosynthesis, it constructs a seamless bio-electronic interface, thereby fundamentally avoiding host immune recognition. The following will systematically dissect the technical paths of these three strategies and clarify their advantages and disadvantages, applicable scenarios, and translational boundaries.

### 2.2 Bionic ECM

Biomimetic ECM material is a strategy that can simultaneously achieve dual functions of inhibiting acute inflammation and integrating with the neuron electrode interface. It is also a technical system with sufficient in vivo validation data for FBR regulation.

**Fig. 3:**
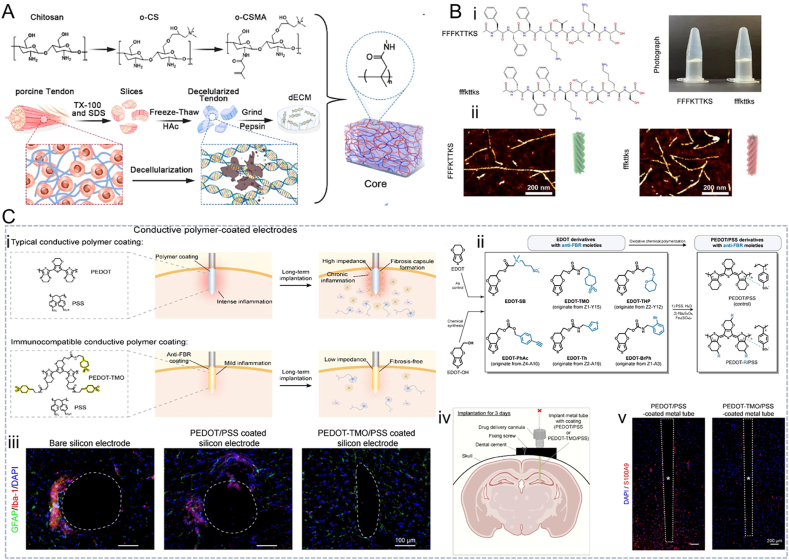
(A) Fabrication of Core-Sheath Nerve Conduits (NGCs). Structure: A “core-sheath” design featuring a ZnO@PLLA nanofibrous sheath and a QCS-MA/dECM hydrogel core. Reproduced with permission [61]. Copyright 2024, Elsevier. B.V. (B) Characterization of Peptide Hydrogels. i. Sequence: Molecular structure of collagen-derived peptides with biomimetic ECM activity. ii. Morphology: AFM images showing the self-assembled structures of FFFKTTKS and fffkttks. Reproduced with permission [62]. Copyright 2025, Wiley VCH. (C) Conductive Polymer Coatings and Brain Tissue Response. i. Concept: Immunocompatible PEDOT coatings on silicon electrodes to improve long-term neural recording performance. ii. Chemistry: Synthesis and screening of PEDOT derivative monomers and their polymerization. iii. Histology: Immunofluorescence comparing tissue response to coated vs. uncoated electrodes 4 weeks post-implantation. iv. Model: Schematic of a metal drug delivery tube implanted in the rat brain. v. Staining: Immunofluorescence images of S100A9 (red) and DAPI (blue) expression (n = 3). Reproduced with permission [63]. Copyright 2025, American Chemical Society.

Tissue-mimetic bioelectronics are engineered to establish seamless integration between neuroelectronic devices and host brain architectures via the construction of bioactive interfaces, while concurrently modulating immune responses [40]. The extracellular matrix (ECM)—a pivotal acellular component of native tissues—does not merely serve as a structural scaffold for cellular populations, but also exerts a central role in sustaining immune homeostasis within the central nervous system (CNS) [56,57]. In the CNS, the ECM constitutes approximately 10–20% of the total brain parenchymal volume. Its distinctive biochemical composition and hierarchical architecture mediate cellular adhesion, migration, and functional phenotypic expression through integrin-dependent signaling pathways, thereby exerting critical regulatory effects on neural development, synaptic plasticity, and tissue reparative processes. Given that aberrant ECM remodeling is closely correlated with a spectrum of neuropathological conditions [58], the fabrication of neural electrode interfaces via the biomimetic recapitulation of native ECM bioactivities represents a promising strategy to mitigate implant-induced immune responses. To date, such ECM-mimetic strategies have demonstrated broad application potential across diverse biomedical fields, including cartilage regeneration guidance [59], chronic wound healing promotion [60], and neural repair enhancement (Fig. 3A) [61].

Before surveying specific strategies, it is useful to clarify two concepts that recur throughout this review. By “immune homeostasis-maintaining capacity,” we refer to the ability of an electrode surface to actively shift the local glial balance away from a pro-inflammatory, neurotoxic phenotype and toward a reparative, anti-inflammatory state. This is achieved not through systemic immunosuppression, but through locally presented chemical cues (e.g., THP/TMO side chains) or topographical signals that polarize adherent microglia and macrophages toward the M2 phenotype. The closely related concept of “metabolic adaptability” describes a material's capacity to sense and neutralize local biochemical stress signals, most notably reactive oxygen species released by damaged tissue, through built-in antioxidant functionalities such as selenium-containing backbones or ceria-based coatings. In both cases, the functional moieties are covalently immobilized on the electrode surface, ensuring that their immunomodulatory action remains spatially confined to the peri-prosthetic microenvironment and does not perturb healthy brain parenchyma. These two principles, acting at the interface of chemistry and biology, form the conceptual basis for the active immunomodulatory strategies discussed below.

In vivo, the activation status and population density of microglia and astrocytes surrounding biomaterials are widely regarded as hallmark indicators for evaluating immune rejection and biocompatibility mismatch [45,64]. The successful integration of biomaterials hinges on a dynamic equilibrium between the initiation of inflammatory responses and their timely resolution [65], with the latter being an indispensable prerequisite for effective tissue repair. Although the complete elimination of host immune responses to implants is not clinically feasible, minimizing the extent of fibrosis and inflammation at the implantation site is pivotal for facilitating biological integration and functional tissue regeneration. In the design of neural electrode interfaces, fabricating functional interfaces that recapitulate the physical architecture of the native ECM has emerged as a pivotal strategy for enhancing electrode biocompatibility and suppressing immune activation. Hydrogels have emerged as ideal biomimetic platforms owing to their ability to closely mimic the three-dimensional microenvironment and biochemical signaling cues of the native ECM. These water-swellable crosslinked polymer networks possess high water content and mechanical flexibility that closely resemble native tissues, while recapitulating the 3D structural features of the natural ECM within the central nervous system (CNS) [66]. For instance, the immobilization of functional hydrogel coatings onto conventional metal electrode surfaces—such as ECM-mimetic soft hydrogel coatings based on polyethylene glycol (PEG) [67] and polyvinyl alcohol (PVA) [68,69], grafted onto rigid electrode substrates—are associated with reduced glial scar formation and neuronal loss, which correlates with alleviated implant-induced neuroinflammatory responses [70]. Further in vivo experiments verified that, compared with uncoated platinum (Pt) electrodes or PEDOT:PSS coatings, the PEDOT:Poly(SS-4VP) coating markedly attenuated GFAP expression and reduced the accumulation of reactive astrocytes in the peri-implant region. The superior stability and high surface roughness of this hydrogel coating provide a favorable interface for neural cell adhesion and differentiation, consequently leading to a significant reduction in host immune responses [71].

Secondly, ECM-based materials can be used directly as biomimetic surrogates for the native brain matrix, thereby enhancing cellular adhesion to neural electrodes. Furthermore, ECM-mimetic materials have been shown to possess hemostatic and immunomodulatory properties [72,73], with these functionalities corroborated in clinical settings. Thus, ECM-derived proteins—including laminin, collagen [74] , and its derivative gelatin [75]—represent promising candidates for the surface modification of neural electrode interfaces. In the field of neural electrode interfaces, a prevalent strategy involves immobilizing ECM components onto electrode surfaces either through covalent conjugation or physical adsorption. For instance, researchers successfully fabricated an astrocyte-derived ECM bioactive coating and applied it to the surface of penetrating neural microelectrodes [76]. This coating exhibited robust procoagulant activity, which effectively expedited the hemostatic cascade. Following implantation into the cerebral cortex of adult rats, the ECM-modified electrodes elicited a marked reduction in astrocyte proliferation within the peri-implant region, in sharp contrast to unmodified control electrodes. Notably, during the 8-week implantation period, both the spatial extent and intensity of GFAP immunoreactivity were significantly diminished, confirming the coating's sustained inhibitory efficacy against astrocyte activation. Further proteomic profiling revealed that astrocyte-derived ECM possesses anti-scarring and anti-inflammatory bioactivities and can potently modulate macrophages activation status. Consequently, this coating is associated with mitigated chronic foreign body reactions (FBRs) throughout the long-term implantation phase, thereby furnishing a critical safeguard for the functional stability of neural electrodes.

Neuronal loss and reactive astrogliosis triggered by inflammatory responses in brain tissue are pivotal factors underlying the degradation of recording performance and the shortened lifespan of neural electrodes [77,78]. Beyond physicochemical biomimetic design, the surface functionalization of electrodes with bioactive moieties that recapitulate the biochemical microenvironment of host tissues—including ECM-derived proteins, cell adhesion molecules, and growth factors—enables more comprehensive promotion of interfacial integration [79]. To address this critical issue, researchers have explored diverse strategies for electrode modification using bioactive molecules. For instance, the adsorption of laminin onto the surface of polypyrrole/p-toluenesulfonate (PPy/pTS) films has been shown to significantly promote neurite outgrowth [80]. Similarly, the self-assembly of collagen into a three-dimensional biomimetic matrix effectively enhances the adhesion and differentiation of neural cells, thereby improving the interfacial integration of electrodes [81]. Neural cell adhesion molecule L1—a neuron-specific protein highly expressed in the central nervous system (CNS)—exhibits considerable potential in facilitating neurite outgrowth, axon guidance, and neuronal survival [82,83]. When neurons rapidly and stably adhere to and envelop the electrode surface, an interface resembling native tissue is formed, minimizing the electrode's exposure as a foreign body. Following the covalent immobilization of L1 onto silicon-based neural probes and subsequent long-term in vivo observation after implantation into the rat cortex [82], results demonstrated that, in contrast to unmodified probes, there was no significant neuronal loss within the 100 μm peri-implant region surrounding L1-modified probes; axon density even exhibited a relative increase compared to the background, while the immune responses of microglia and astrocytes were markedly attenuated. Further investigations have verified that the L1 coating can directly modulate microglia inflammatory behavior [84]. Under lipopolysaccharide (LPS) stimulation, L1 was found to significantly downregulate the expression of inducible nitric oxide synthase (iNOS), reduce nitric oxide (NO) production, and suppress the secretion of pro-inflammatory cytokines (TNF-α, IL-6, and IL-1β) in microglia. Concurrently, it upregulated anti-inflammatory cytokines (TGF-β1 and IL-10), enhances microglial phagocytic activity, and promotes Arg-1 expression, indicating its potential to facilitate inflammatory resolution and tissue repair. In addition, L1 can effectively mitigate macrophage chronic activation and prevent neuronal loss in the tissue surrounding implanted microelectrode arrays. Beyond L1, bioactive molecules containing cell adhesion sequences—such as the arginine-glycine-aspartic acid (RGD) motif [85] —can also improve cell-electrode material interactions, promoting tight cellular adhesion to the electrode surface and forming a physical immune barrier.

Chakraborty et al. designed a de novo-designed peptide hydrogel, Fmoc-K(Fmoc)-RGD (where K = Lys), that intrinsically incorporates the RGD motif. By introducing polyaniline (PAni) to nanoengineer the peptide hydrogel network [86], they constructed a composite hydrogel with semiconducting properties [87], which exhibited excellent antibacterial activity and DNA-binding capacity. Compared with full-length proteins, peptide fragments possess superior stability, are less susceptible to rapid degradation, and have a lower propensity for inactivation. Typically, researchers derive specific bioactive fragments from native proteins, including laminin, fibrin, and collagen. Self-assembled hydrogels fabricated from collagen hydrolysate peptide sequences with biomimetic ECM activity have also been shown to upregulate the expression of ECM-associated genes, while modulating neural cell adhesion, neuronal differentiation, and synaptic regeneration (Fig. 3B). Notably, in animal models of spinal cord injury, ECM-mimetic hydrogels facilitated enhanced neuronal regeneration and motor function recovery, accompanied by a mild immune response and moderate anti-inflammatory activity [62]. Beyond extraction from native proteins, chemical synthesis enables precise control over peptide composition, structure, and purity, allowing the generation of complex sequences tailored for specific biological functions. A 19-mer peptide containing the IKVAV pentapeptide motif has been identified as one of the key domains responsible for regulating cellular behavior in laminin [88]. Farrukh et al. seeded neural progenitor cells onto polyacrylamide (PA) gels conjugated with ε-polylysine (ε-PL) and IKVAV, respectively. Their findings confirmed that PA gels functionalized with both IKVAV and ε-PL could enhance neurogenesis and promote neurite outgrowth [89].

The core advantage of this strategy lies in significantly enhancing electrode biocompatibility by mimicking the biochemical and biophysical signals of the native ECM. Specifically, it can guide neuronal adhesion and axonal growth, inhibit glial scar formation, reduce the foreign body response, and avoid reliance on burst release or the toxicity risks associated with exogenous drugs. However, this strategy still has inherent limitations. Natural ECM proteins are at risk of rapid degradation and insufficient long-term stability (synthetic ECM mimics may be a solution). In addition, completely eliminating the host's immune response to implants is not feasible in clinical practice. Current research mainly focuses on central and peripheral neural interfaces represented by flexible cortical electrodes. Related materials have shown strong application prospects in soft tissue repair, and in vivo validation for neural electrodes is relatively sufficient.

### 2.3 Immune functional polymer

The core value of immune-regulating polymers lies in their ability to modulate multiple inflammatory pathways through precise molecular design. Moreover, they can be combined with conductive polymers, such as PEDOT, to form a neural electrode interface that combines excellent electrochemical performance with long-term biocompatibility.

Immunomodulatory polymers have demonstrated substantial potential for neural interfaces, enhancing the biocompatibility and long-term stability of implanted electrodes by regulating immune responses. Natural materials such as silk fibroin and cellulose are utilized to improve electrode biocompatibility; for instance, silk fibroin-coated cortical electrodes have been shown to reduce the expression of glial hyperplasia markers [90,91]. Further studies have indicated that biomolecule-modified conductive polymers can enhance interactions at the neuron–electrode interface while mitigating inflammatory responses [92]. For example, cross-linking silk fibroin with polyethylene glycol diglycidyl ether (PEGDE) and depositing the conjugate onto PEDOT:PSS films enables the fabrication of transparent, stretchable, and electrochemically stable gel electrodes. These electrodes establish robust electrical coupling with brain tissue and facilitate in situ recording of neurovascular activitie [93]. Furthermore, conductive hydrogels with immune-evasive properties (dPEDOT-CA-PDA-PAM) have been successfully developed by incorporating carrageenan (CA) into an interpenetrating polydopamine-polyacrylamide (PDA-PAM) network, using dopamine methacrylate (DMA)-hybridized PEDOT nanoparticles as the conductive component. In vitro experiments demonstrated that this hydrogel can effectively modulate immune cell behavior, significantly downregulating the expression of the pro-inflammatory cytokine TNF-α in macrophages while concurrently upregulating the levels of the anti-inflammatory cytokine IL-10. In vivo experiments further validated its superior immunomodulatory capabilities: two weeks after subcutaneous implantation in Sprague–Dawley (SD) rats, negligible inflammatory responses were observed in the peri-hydrogel tissues. Immunofluorescence staining revealed a significant reduction in the infiltration of CD3^+^ T cells and CD68^+^ macrophages, along with decreased expression of the fibrosis markers α-smooth muscle actin (α-SMA) and type I collagen [94]. These findings indicate that the dPEDOT-CA-PDA-PAM hydrogel can effectively downregulate pro-inflammatory cytokines, inhibit the formation of fibrotic encapsulation, and establish a long-term, stable immune-privileged microenvironment at the implantation interface. Compared with conventional PEDOT:PSS, PEDOT-TMO (transition-metal-oxide-doped PEDOT) is a promising candidate with excellent biocompatibility and robust electrical conductivity. Following subcutaneous implantation in mice, PEDOT-TMO significantly reduces acute inflammatory and chronic fibrotic responses while preserving its intrinsic electrochemical performance. In long-term in vivo brain implantation studies conducted in freely moving rat models, PEDOT-TMO/PSS-coated silicon electrodes reliably recorded electrophysiological signals for at least 8 weeks, substantially extending the long-term implantation stability of bioelectronic devices. The core mechanism is that the PEDOT-TMO/PSS interface inhibits neuroinflammation by targeting and regulating surrounding brain tissue (Fig. 3C) [95].

Although research on the mechanisms underlying the interaction between material chemical structures and the immune microenvironment is still evolving, advancements in immunomodulatory materials science have uncovered novel design strategies. For instance, selenium and its derivatives (e.g., selenocysteine) can regulate macrophage polarization by scavenging reactive oxygen species (ROS), while specific side-chain groups (e.g., THP/TMO) can directly interfere with inflammatory signaling pathways. Building on this, researchers adopted an innovative molecular design strategy: via microwave-assisted polymerization, they synthesized a backbone structure p(g2T-Se) in which traditional thiophene (T) was replaced with selenophene (Se). Additionally, the click-to-polymer (CLIP) method was employed to achieve 100% grafting efficiency of the side-chain THP/TMO groups. Four weeks post-implantation, the collagen deposition density of p(g2T-Se)-THP was significantly reduced. The selenophene backbone decreased the mRNA expression levels of type I and type III collagen by 20–40%, while THP/TMO side-chain functionalization further reduced these expression levels to 50–70% lower than the baseline. The optimized p(g2T-Se)-TMO material significantly inhibited macrophage infiltration and myofibroblast formation, and effectively blocked the fibrotic progression of the foreign body response (FBR). The synergistic effect of selenophene and THP/TMO upregulated the expression of anti-inflammatory factors, drove macrophage polarization toward the reparative M2 phenotype, and reshaped the immune microenvironment. Furthermore, the modified selenophene significantly inhibited key inflammatory signaling molecules (e.g., p-p38, p-JNK) in LPS-activated macrophages in a dose-dependent manner, and interfered with the innate immune response by scavenging ROS [96]. However, immune cell infiltration remains an inherent challenge for such interfaces. A research team proposed an innovative strategy to achieve robust interfacial anchoring via bioadhesive and conductive hydrogels. This “fibrosis-free bioelectronic interface” was integrally assembled via 3D printing using multiple materials: a conductive PEDOT:PSS layer, a polyurethane insulating layer, and a PVA/PAA adhesive hydrogel layer. It exhibits excellent flexibility, stretchability, and electrical conductivity. This adhesive layer can form covalent bonds with the neural tissue surface, thereby preventing immune cell infiltration and reducing fibrous capsule formation and chronic inflammatory responses. Researchers successfully achieved 12-week implantation without fibrotic encapsulation across various neural interfaces in rats (including the occipital nerve, vagus nerve, sciatic nerve, and common peroneal nerve), completely blocking the fibrotic progression of the neural interface following electronic device implantation [97,98].

The core advantage of this strategy lies in its ability to precisely customize material properties, enabling multi-target immune regulation (such as eliminating ROS, driving macrophage polarization towards a repair phenotype, and inhibiting collagen deposition and fibrosis progression). Furthermore, it maintains good electrochemical performance even after electrode modification. However, the mechanism underlying the interaction between the material's chemical structure and the immune microenvironment remains poorly understood, and related research is still evolving, limiting the precision and predictability of material design. Currently, this strategy is better suited to applications such as silicon-based intracranial microelectrodes and peripheral nerve interfaces.

### 2.4 Biohybrid electronics

Biohybrid electronics is the frontier disruptive strategy of FBR regulation in the acute phase, which breaks away from the idea of traditional material modification and fundamentally eliminates the host's recognition of foreign bodies in implants.

Biohybrid electronics enables the construction of authentic, seamless, and multifunctional biological interfaces with tissues by synergistically integrating biomechanical, bio-derived, and bioelectrical properties. Electrode devices with interfaces based on direct conjugation of bioactive molecules to existing electronic materials have demonstrated substantial potential for eco-friendliness, cost-effectiveness, and novel computing principles [99]. Biohybrid devices specifically refer to structures in which electrode materials are “encapsulated” by living cells [100]. Their origins can be traced back to brain–computer interface (BCI) research in the early 1990s, and they have regained attention with advances in flexible electrode materials. Among these, cell-seeded electrodes represent a key application direction of biohybrid electronics. By directly seeding living cells onto electronic devices or encapsulating them within hydrogel scaffolds, the device-tissue interface can be enhanced, facilitating tissue regeneration and functional recovery [101].

Once the cells within these biohybrid electronic devices establish an interface with the host tissue, they can markedly enhance biological integration and hold promise as active scaffolds for probing pathophysiological processes or facilitating tissue regeneration. This approach synergizes the merits of tissue engineering and bioelectronics by constructing an in-device biological platform, thereby improving biocompatibility and long-term integration performance. Leveraging the innate capacity of neurons to form non-invasive connections, this strategy enables atraumatic integration with the brain.

**Fig. 4:**
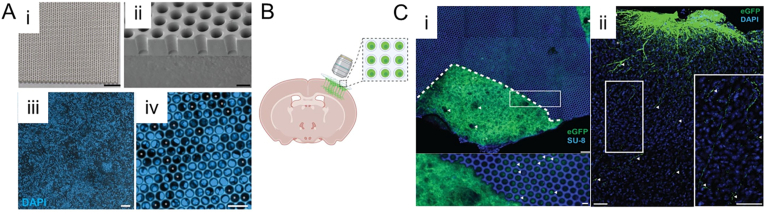
(A) Microwell Scaffold Characterization. i-ii. Imaging: Light micrograph of a scaffold on a coverslip (i) and a 45° tilted SEM image of an empty gold-sputtered microwell (ii). iii-iv. Cell Loading: Low and high-zoom confocal images of DAPI-stained cells in microwells 24 h post-loading. Asterisks mark empty wells. (B) Experimental Design. Setup: Schematic showing mouse primary neurons labeled with AAV-CheRiff-eGFP, loaded into scaffolds, and implanted onto the mouse brain surface. (C) Post-Implantation Analysis (Months Later). i. Explant Histology: Confocal image of an explanted scaffold. White dashed lines indicate fractured regions where brain tissue (including blood vessels, white arrows) remained adhered to the graft. The inset shows cells still residing within microwells. ii. Cortical Integration: Max-projection confocal image of the cortex beneath the explant site. Shows CheRiff-eGFP + processes in the superficial cortex and putative axons (white arrows/inset) deeper in the brain. Reproduced with permission [102]. Copyright 2026, bioRxiv.

Specifically, neurons were cultured in the microwells of a waffle-like microporous device, which was then inverted and placed onto the brain surface. These neurons subsequently extended downward into the brain parenchyma, serving as biological linkers bridging the device and brain tissue. In a proof-of-concept experiment, neurons derived from embryonic stem cells of isogenic mouse lines were cultured and then implanted into recipient mice together with the device for in vivo validation. The waffle-structured device featured a transparent substrate patterned with microwells approximately 10 μm in diameter, each containing a single neuron (Fig. 4A–B). Each 5 mm × 5 mm device could accommodate an average of 90,000 neurons. Experimental results demonstrated that neurons in the biohybrid device migrated to the outermost layer of the cerebral cortex, while neovascularization was also observed within the newly formed neuronal network. Fluorescence imaging of brain tissue beneath the implant further confirmed that neuronal axons successfully penetrated the superficial dense cell layer and innervated the first layer of the cerebral cortex [102] (Fig. 4C).

**Fig. 5:**
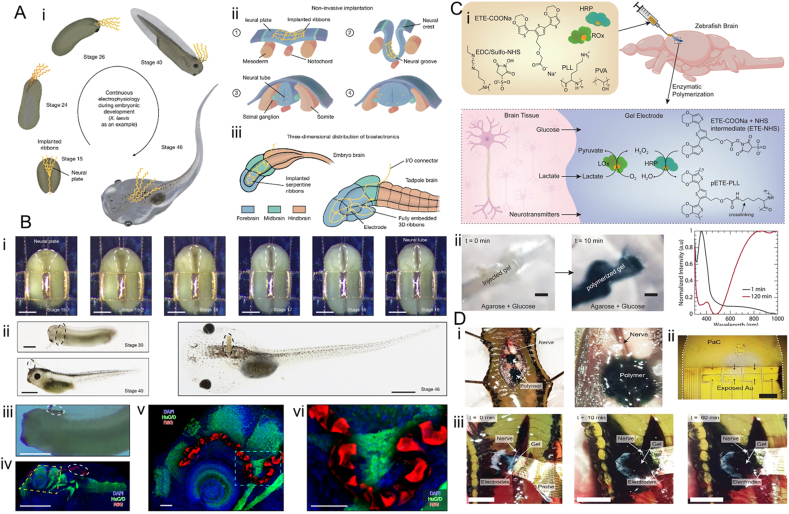
(A) Integration of Mesh Electronics via Organogenesis. i-ii. Process: Schematics of soft mesh electronics are laminated onto the neural plate (Stage 15) and naturally internalized into the 3D neural tube (Stages 24–46) during embryonic folding. iii. Connectivity: The stretchable mesh expands with the brain's 3D growth, maintaining an external connection via an I/O connector. (B) Morphological and Fluorescence Imaging. i-ii. Development: Bright-field time-lapse and photos (Stages 30–46) showing the gradual internalization of the electrode array (dashed circles). iii-iv. 3D Visualization: Photograph and 3D confocal reconstruction of a “cyborg tadpole” stained for nuclei (DAPI), the device (R6G), and neurons (HuC/D). v-vi. Interface: High-magnification views showing the mesh folded within the neural tube and its intimate interface with embedded neurons. Reproduced with permission [103]. Copyright 2025, Springer. (C) In Vivo Polymerization Mechanism. i-ii. Validation: Schematic and demonstration of “cocktail gel” injecting into glucose-containing agarose, turning dark blue upon polymerization (10 min). Spectra: UV-vis measurements confirming the chemical transition from 1 to 120 min post-injection. (D) Flexible Probes and Bio-induced Gels. i. Animal Model: Polymerized lactate-induced gels surrounding a leech's central nerve. ii. Device Structure: Microscopic view of a PaC-based probe with eight Au electrodes and insulated interconnects (Scale bar: 500 μm). iii. Gel Formation: Color change (clear to dark blue) of the conducting gel on the flexible probe over 1 h. Reproduced with permission [104]. Copyright 2023, American Association for the Advancement of Science.

From biohybrid living-cell electronics to direct in vivo biosynthesis, researchers continue to push the boundaries between engineered materials and biological organisms. Although advances in flexible electronics have enabled tissue-like mesh microelectronic devices to achieve seamless integration with neural tissues both in vitro and in vivo, supporting long-term, stable multichannel electrophysiological recordings, implanting of such devices into the mature brain inevitably disrupts the blood–brain barrier (BBB), triggering acute immune responses. To address this challenge, one research group has pioneered a novel implantation strategy that capitalizes on the natural tissue morphogenesis occurring during embryonic development to facilitate bioelectronic device integration. Using confocal fluorescence microscopy imaging, the researchers confirmed that the devices were successfully incorporated into multiple brain regions, including the forebrain, midbrain, and hindbrain. Tight electrode–neuron contacts were visualized, indicative of favorable device–neural tissue integration. Notably, embryos implanted with the devices showed no significant differences from non-implanted control embryos in cell proliferation, differentiation, or immune responses, demonstrating that device implantation did not adversely affect embryonic development. Leveraging the implanted devices for long-term neural activity monitoring, the team successfully recorded neural signals spanning the embryonic to tadpole stages, including slow oscillations, calcium wave-like signals, and single-unit action potentials. These recordings delineate a dynamic transition process, in which neural activity evolves from global brain-wide synchronous oscillations to localized neural activity, and ultimately to the emergence of single-unit action potentials [103] (Fig. 5A–B). The development of this next-generation bioelectronic device aims to enable long-term, stable, high-resolution neural activity recordings from the embryonic to mature stages. Concurrently, it seeks to validate the device's impact on brain development and function to ensure the absence of deleterious effects on embryonic development.

Furthermore, via in situ biopolymerization technology, researchers have ingeniously harnessed the intrinsic metabolic networks of living organisms to achieve the controlled growth of conductive polymers. This technique employs lactic acid molecules as the “fuel” and natural enzymes as the “catalysts”, operating entirely under physiological conditions. When this “electronic ink” is injected into biological tissues, lactic acid molecules diffuse into the hydrogel matrix and are oxidized by lactate oxidase (LOx) to generate hydrogen peroxide (H_2_O_2_). Horseradish peroxidase (HRP) then utilizes H_2_O_2_ to catalyze the oxidative polymerization of ETE monomers. Simultaneously, the amino groups of poly-L-lysine (PLL) form stable covalent linkages with activated ETE-NHS intermediates, thereby constructing a 3D network with integrated electrical conductivity and mechanical elasticity. Notably, the entire process is driven exclusively by the organism's endogenous metabolism, eliminating the need for external energy input. Using two-photon microscopy, the researchers captured, for the first time, the full dynamic process of conductive polymer formation within the living nervous system. At 72 h post-polymerization, no overt inflammatory responses were detected in the fish brain (Fig. 5C–D). These in vivo-synthesized conductive networks also exhibit a unique “adaptive” characteristic: when elevated neural activity in surrounding tissues increases in lactic acid concentration, the polymer network undergoes further extension and growth. Additionally, the in vitro polymerization of the hydrogel was induced at the interface between neural tissue and gold electrodes to reduce interfacial impedance [104].

In conclusion, enhancing biocompatibility is a crucial step in controlling the early acute immune response after neural electrode implantation. Strategies focused on ECM-mimetic interfaces, biohybrid electronics, and immunomodulatory polymer engineering have led to significant progress. These advances help reduce inflammation, suppress foreign body reactions (FBR), improve neuronal viability, and stabilize signals. Looking ahead, future electronic devices may be designed to actively incorporate themselves into host tissues, growing and adapting alongside biological structures. In this scenario, bio-self-assembled electronics will exist in a dynamic, intermediate state, facilitating sustained interaction and adaptation between humans and machines. This vision of co-growth and symbiosis represents a shift from current models, suggesting that human–machine integration could become a process of mutual development rather than mere combination.

Turning to the underlying fundamental scientific issues, inadequate modulation of acute-phase inflammation can impair the integrity of local vasculature and compromise immune clearance capacity, thereby creating a permissive microenvironment for pathogen colonization. Such dysregulation of the immune microenvironment not only elevates the risk of secondary infections but also drives the progression of the FBR toward chronicity and fibrosis, ultimately culminating in the functional failure of the electrode–tissue interface. Thus, building on the initial achievements in immune modulation, maintaining the long-term stability of neural interfaces necessitates addressing another critical challenge: preventing persistent chronic inflammation triggered by microbial colonization and biofilm formation. Bacterial biofilms can perpetually activate the host immune system, exacerbate tissue damage, and precipitate a vicious cycle of aggravated FBR. In this context, the construction of multifunctional intelligent interfaces with integrated antifouling and antibacterial properties—while preserving fundamental biocompatibility—is of profound significance for blocking secondary infections and sustaining immune microenvironment homeostasis.

The core advantage of this strategy lies in its natural integration with the host neural circuitry, which triggers only minimal acute inflammatory responses and effectively blocks the initiation of FBR. However, its inherent limitations include unpredictable survival time in living cell systems, the risk of tumor formation, regulatory issues, and potential incompatibility with clinical deep-brain implantation procedures. Therefore, this strategy is suitable for developmental implantable electrodes in model animals and is currently only at the proof-of-concept stage in small animals, with no short-term clinical translation.

Based on the current research progress on the three strategies, there are still two key bottlenecks in the field of acute-phase FBR regulation guided by core scientific principles. Firstly, the causal relationship between acute-phase inflammation regulation and long-term electrode stability has not been rigorously verified. From the perspective of the pathological cascade principle of FBR, although acute-phase inflammation is one of the initiating factors for long-term electrode failure, the final interface fibrosis and signal attenuation are driven by multiple factors. Many modification strategies simultaneously alter the mechanical properties, surface chemistry, conductive characteristics, and anti-inflammatory functions of materials, making it difficult to confirm through univariate targeted experiments that acute-phase inflammation inhibition is the direct cause of improved long-term stability. This may be an important scientific root cause for the short-term effectiveness and long-term failure of some materials. Secondly, the combination of multiple strategies presents timing conflicts, violating the pathophysiological evolution law of acute-phase FBR. Acute-phase FBR has distinct subphases with completely opposite functional requirements: the core requirement in the hyperacute phase is to block the initiation of the inflammatory cascade, whereas in the later stage it is to promote neural interface integration. Existing research almost simply superimposes and synchronizes the anti-inflammatory and neuro-integration-promoting functions, ignoring the issue of timing matching. Specifically, early-released regenerative-promoting molecules will rapidly degrade and lose efficacy in the inflammatory microenvironment, while continuously released broad-spectrum anti-inflammatory drugs may interfere with the normal physiological functions of glial cells in tissue repair and neural interface integration. To address this issue, this review proposes a phased functional design based on timing as a solution direction.

## 3 Long-term antifouling and antibacterial interface design

Given the core pathological challenges of biofouling and bacterial biofilm formation during the chronic implantation stage of nerve electrodes, antifouling and antibacterial measures are common core strategies for preventing and controlling biofouling in implantable bioelectronic devices. Related mature technologies have been widely used in cardiovascular stents, cochlear implants, and other implantable devices, and have a scientific basis for optimizing the design of brain-computer interface electrodes. The existing solutions are divided into two complementary technical directions, corresponding to the two core links: initial adhesion blocking and post-colonization biofilm sterilization. The antifouling strategy is divided into three core systems: zwitterion coating, anti-adhesion lubrication coating and dynamic polymer brush. The three systems achieve antifouling through completely different interface-chemistry mechanisms. Antimicrobial strategies can be divided into two categories: antimicrobial peptide chemical sterilization and micro- and nanoscale physical sterilization. The core difference is whether there is a risk of bacterial resistance. The following will systematically disassemble the technical paths of various strategies and the feasibility of cross-domain migration, and discuss their advantages, disadvantages, and application boundaries in the future intracranial implantation scenario of brain-computer interface.

### 3.1 Antifouling surface design

**Fig. 6:**
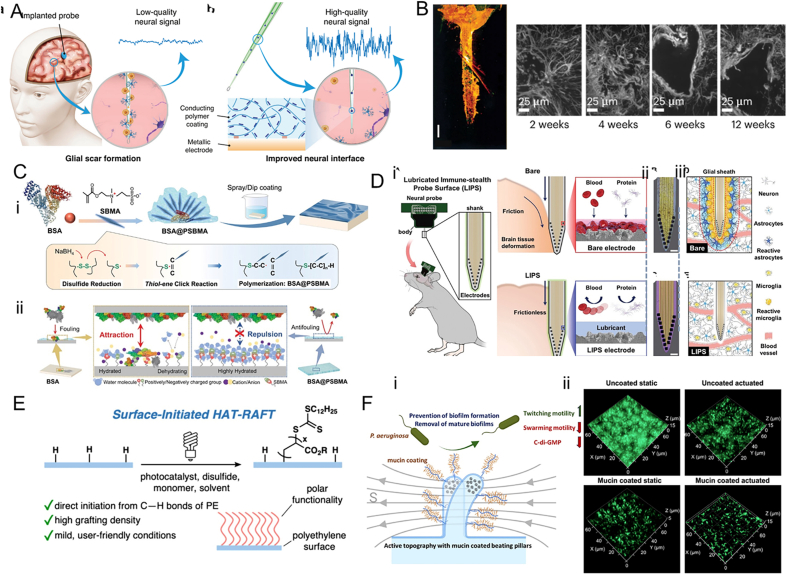
(A) Conductive Hydrogel Coatings for Electrodes. Mismatch Issue: Schematic showing how a mechanical/electrical mismatch between metal electrodes and brain tissue causes scar formation and high impedance. Hydrogel Solution: Illustration of a PEDOT:PSS hydrogel coating that enhances biocompatibility, reduces impedance, and improves stimulation/recording efficacy. Reproduced with permission [71]. Copyright 2023, Wiley VCH. **(B)** Glial Scarring Characterization. Chronic Response: The top micrograph shows a silicon probe covered in astrocytes (GFAP + staining) 6 weeks post-implantation. Time Course: Cross-sectional images (bottom) tracking the 6-week progression of glial scar formation around a cortical implant. Reproduced with permission [8]. Copyright 2023, Springer. **(C)** BSA@PSBMA Synthesis and Antifouling. **i**. Synthesis: Schematic of BSA@PSBMA fabrication via thiol-ene click chemistry. **ii**. Force Measurement: SFA measurements of interfacial interactions between foulants and the coating to demonstrate antifouling properties. Reproduced with permission [107]. Copyright 2023, Wiley VCH. **(D)** Lubricated Immune-Stealthy Probe Surface (LIPS). **i**. Mechanism: Comparison of the immune-evasive mechanism between LIPS-coated and bare probes. **ii**. Blood Compatibility: Micrographs of probes after 30-min blood immersion; LIPS shows minimal fouling compared to bare surfaces. **iii**. Encapsulation: Schematic showing the reduction of glial encapsulation on LIPS-treated probes. Reproduced with permission [108]. Copyright 2021, Wiley VCH. **(E)** High-Density Polymer Brushes. Synthesis: Illustration of surface-initiated HAT-RAFT polymerization to create dense polymer brushes directly from PE surfaces. Reproduced with permission [109]. Copyright 2024, American Chemical Society. **(F)** Biofilm Control via Active Topography. **i**. Mechanism: Schematic of mucin-coated “beating pillars” that use physical force and biological signaling to inhibit bacterial biofilm formation. **ii**. Performance: Quantitative data showing enhanced fouling control on actuated pillared surfaces with mucin coating under a 10 Hz magnetic field. Reproduced with permission [110]. Copyright 2025, Wiley VCH.

Anti-fouling coatings are the precondition for biofilm prevention and control. By physically and chemically modifying the interface, they block the initial adhesion of non-specific proteins to bacteria, thereby cutting off the foundation for biofilm formation at its source. This chapter presents the material strategies for the chronic biofouling and infection phase, which is the intermediate regulatory link of the temporal stage-specific design paradigm. Upon implantation into the body, the implant surface rapidly adsorbs proteins from blood and interstitial fluid, forming a transient protein–cellular matrix complex [105]. These protein matrices serve as the primary basis for immune cell “recognition” of the implant. In particular, the adsorption of abundant pro-inflammatory proteins (e.g., fibrinogen) onto the surface activates inflammatory cells [106], thereby triggering the foreign body response (FBR) induced by biofouling (Fig. 6). Although the FBR is initiated instantaneously upon implantation, its progressive accumulation persists over an extended period (ranging from several days to weeks). Thus, targeted interventions should be implemented subsequent to the resolution of the acute immune phase [111]. Anti-fouling coatings are associated with reduced fouling adsorption and reduced the amplification of inflammatory signaling cascades. Distinct from the non-specific protein adsorption occurring in the initial stage, biofouling in the subsequent phase involves the adhesion of exogenous substances—including bacteria, biomacromolecules (e.g., proteins/carbohydrates), cells, and suspended particles—to the sensing interface via reversible or irreversible interactions. Its core components include activated glial cells, cellular debris, and a substantial amount of extracellular matrix (ECM) proteins synthesized and secreted by these cells. Fouling adhesion correlates with bio-contamination, device passivation, and reduced sensitivity, but also elicits false-negative or false-positive signals. Furthermore, it forms functional physical/biochemical barriers (e.g., glial scars and fouling layers), which are associated with the persistence of chronic inflammation and the attenuation of electrode signals [112](Fig. 6A and B). Conventional anti-fouling strategies rely on lowering surface energy to achieve super hydrophobicity and reduce adhesion [[113], [114], [115]]. However, their anti-fouling efficacy is typically attributed to the microscale air layer trapped at the interface. This air layer is prone to depletion following long-term immersion in aqueous environments, and may even exacerbate subsequent microbial adhesion compared to unmodified substrates [116]. Consequently, hydrophobic interfaces exhibit inherent limitations for application in implantable electronic devices; moreover, extreme hydrophobicity can impede tissue integration and elicit adverse immune reactions. In light of these drawbacks, researchers have shifted their focus toward novel biocompatible anti-fouling strategies based on physical barrier mechanisms. Currently, three primary physical interface engineering strategies are employed to achieve efficient, long-lasting, and biocompatible anti-fouling performance: (i) Zwitterionic surfaces: Leveraging strong hydration to construct a dynamic hydration layer at the interface, which effectively repels biomolecule adsorption; (ii) Lubricant-infused bioinspired interfaces: Establishing a stable slippage layer to facilitate the easy desorption of foulants, thus maintaining interface cleanliness; (iii) Dynamic molecular brushes: Harnessing the conformational entropy of polymer chains to resist fouling adhesion, thereby providing long-term anti-fouling capability. These strategies provide pivotal solutions for addressing fouling challenges in complex biological microenvironments, such as those encountered by implantable devices [117].

#### 3.1.1 Zwitterionic antifouling coatings

Zwitterionic polymers, such as sulfonobetaine (SB) [118,119], possess equimolar amounts of cationic and anionic moieties, with these moieties spatially proximal within a single molecular chain. This unique structural feature endows them with exceptional water-binding capacity, enabling the formation of a dense, highly dynamic hydration layer at the material interface [120]. Through synergistic steric hindrance and electrostatic repulsion effects, this hydration layer effectively impedes the non-specific adsorption of biofoulants (e.g., proteins and bacteria). Consequently, it exerts robust inhibitory effects on thrombosis and bacterial colonization, while simultaneously improving the baseline stability of neurotransmitter detection [121,122]. This mechanism serves as the core physicochemical basis for biocompatible anti-fouling strategies, laying a solid foundation for the development of next-generation, high-performance, non-toxic, and anti-fouling materials.

Based on the aforementioned mechanism, various zwitterionic materials have been employed as interface modification agents to enhance the anti-fouling performance of biosensing interfaces. Carbon-based materials (e.g., carbon nanotubes, CNTs) are generally regarded as non-cytotoxic toward neuronal cells. However, their direct exposure to human tissues has been shown to induce aberrant activation of immune cells [123]. To address this issue, researchers have functionalized the surfaces of conductive carbon materials with zwitterions such as poly(sulfobetaine methacrylate) (PSBMA) [124] and phosphorylcholine [125]. This surface modification reduced the membrane contact angle significantly to 15.7° ± 1.5° and drastically diminished protein adsorption [126]. Following a 14-day incubation in human plasma, the impedance of the resulting conductive devices exhibited no discernible alterations, while high sensitivity, a broad linear range, and a low limit of detection toward target analytes were retained [127]. These findings underscore the critical role of anti-fouling performance in maintaining device stability within complex biological microenvironments. In addition, electrodes fabricated from zwitterionic conductive polymers exhibit low impedance, high charge storage capacity, and large charge injection capability. Post-implantation, these electrodes can effectively suppress scar tissue formation while preserving favorable sensitivity and response kinetics [92]. By engineering neural cell/tissue coupling sites on zwitterionic conductive polymers, specific interactions between the electrode and biomolecules or neural cells/tissues can be achieved, without compromising the anti-fouling properties inherent to zwitterionic electrodes. Taking the conductive polymer poly(3,4-ethylenedioxythiophene):poly(styrenesulfonate) (PEDOT:PSS) as a paradigm, a covalently crosslinked network can be formed by blending PEDOT:PSS with sulfobetaine methacrylate (SBMA), the photoinitiator α-ketoglutaric acid, and the crosslinker polyethylene glycol diacrylate. The protein adsorption on this network interface was as low as 0.95 ± 0.27 μg mL^−1^, and its electrical conductivity remained at 6 S cm^−1^ after immersion in phosphate-buffered saline (PBS) at 37 °C for 14 days. Furthermore, the electrode surface constructed from this photo-initiated crosslinked network can be patterned with high resolution via photolithography, featuring line widths and gap widths of 50 μm each. These characteristics collectively demonstrate exceptional long-term anti-fouling capability and electrochemical stability [128].

For the inherently cationic polymer polypyrrole (PPy), its strong propensity for non-specific protein adsorption necessitates chemical modification to improve anti-fouling performance. Specifically, pyrroleamine is first synthesized by reacting pyrrole with 3-dimethylaminopropyl chloride hydrochloride, which then undergoes a ring-opening reaction with β-propiolactone to yield a zwitterionic pyrrole monomer. The resulting ionic solution is mixed with spike protein, drop-cast onto carbon or gold electrodes, and subjected to electropolymerization via cyclic voltammetry to form a modified coating. The protein adsorption rate on the coated electrodes is substantially lower than that on PPy-coated and bare gold electrodes, with values of 38%, 61%, and 86%, respectively. Following incubation in human serum and saliva, the modified electrodes exhibit minimal changes in electrochemical impedance [129], demonstrating excellent anti-biofouling performance and the ability to mitigate scar tissue formation [130]. This strategy provides a scalable approach for modifying other protein-adsorbent materials.

In contrast to zwitterionic polymers, small-molecule zwitterionic compounds (e.g., 3-aminopropyldimethylamine oxide, APDMAO) feature superior solubility and processability, enabling direct immobilization onto membrane surfaces via molecular grafting. Inspired by the superhydrophilic penetrant trimethylamine oxide found in saltwater fish and the strong adhesive properties of mussels, APDMAO can be utilized to achieve hydrophilic modification of hydrophobic membranes through covalent/non-covalent interactions with dopamine in alkaline buffer solutions. Molecular dynamics (MD) simulations revealed at the atomic scale that APDMAO possesses superior hydrophilicity compared to sulfonated 3-dimethylaminopropylamine (SDMAPA), a zwitterionic agent reported in previous literature. Sum frequency generation (SFG) vibrational spectroscopy further confirmed that the introduction of zwitterionic moieties (-N^+^-O^-^) significantly enhances the surface hydration capability of the modified membranes [131]. In addition, biocompatible biological macromolecules such as bovine serum albumin (BSA) exhibit both surface anchoring capability and anti-fouling performance. However, the surface charge properties of native BSA coatings are susceptible to harsh environmental conditions including high salinity and fluctuating pH values (e.g., post-surgery or during systemic illness), which compromises their anti-fouling functionality [132]. To enhance stability, zwitterionic sulfobetaine methacrylate (SBMA) segments can be grafted onto native BSA molecules via a facile radical-mediated thiol-ene click reaction, yielding the modified conjugate BSA@PSBMA. Benefiting from its biomimetic structure as well as robust interfacial hydration and steric repulsion effects, the BSA@PSBMA coating displays high-efficiency resistance to the adsorption of over 99.9% of biomacromolecules in fetal bovine serum, with anti-fouling efficacy more than 10-fold higher than that of native BSA coatings (Fig. 6C) [133].

Three months after direct implantation of zwitterion-modified hydrogel electrodes, the collagen density around the carboxybetaine (PCB) hydrogel showed a relatively uniform and diffuse distribution. This indicates that the surrounding substance is normal extracellular matrix (ECM) rather than a foreign body response (FBR)-induced capsule. In sharp contrast, poly(2-hydroxyethyl methacrylate) (PHEMA) hydrogel induced extremely high collagen density adjacent to the PHEMA/tissue interface, forming a dense collagenous foreign body capsule. This high-density fibrous capsule not only impedes device signal transmission but also increases the risk of long-term complications, such as mechanical deformation due to capsule contraction [134,135]. Additionally, PCB hydrogels elicit the recruitment of more anti-inflammatory M2-type macrophages and reduce the infiltration of pro-inflammatory M1-type macrophages in the surrounding tissues, thereby promoting neovascularization and tissue remodeling [136]^,^ [137]. This microenvironment polarization effect is achieved through the surface's anti-biofouling properties and the reduced release of inflammatory factors [138].

Overall, the integrated anti-fouling and integration design of zwitterionic polymers at the implantation site provides a new paradigm for the long-term biocompatibility of implantable devices. However, zwitterionic polymers as anti-fouling coatings for electrodes still face an "Achilles' Heel”: all zwitterionic coatings suffer from long-term stability issues in electrolyte environments—the ionic strength of bodily fluids may compromise the hydrated layer that underpins the anti-fouling performance of electrically neutral surfaces [139]. Furthermore, although zwitterions can reduce the adsorption of most proteins, their inherent high hydration properties result in electrode impedance far exceeding the acceptable threshold. Therefore, achieving a balance between excellent anti-fouling performance and acceptable electrode impedance directly affects the overall performance and long-term stability of the electrodes.

#### 3.1.2 Anti-adhesion lubricating coating

The signal sensitivity of implantable devices deteriorates over time, primarily attributable to acute inflammation induced by electrode insertion and chronic inflammation triggered by foreign body reactions. Meanwhile, friction between the electrode and adjacent tissues may further exacerbate the inflammatory response. Therefore, the development of lubricious surfaces is of critical importance, as these surfaces can not only minimize insertion-related friction and reduce fouling adhesion, but also mitigate immunogenicity during neural signal recording [108]. In the early phase of electrode implantation, glycerol was applied to reduce friction between the electrode and brain tissue. The results demonstrated that at 2, 4, and 6 weeks post-implantation, the extent of astrocyte proliferation and reactive gliosis around the implantation site was relatively limited, indicating a mild immune response elicited by device implantation [140]. However, this study employed short-term biodegradable electrodes; from the perspective of long-term application requirements, this single strategy is far from sufficient. To achieve long-term lubrication, researchers have explored various approaches to constructing lubricious interfaces, which can be categorized primarily into ionic-liquid-infused surfaces [141] and liquid-like surfaces [142], based on distinct lubricant-layer retention mechanisms. Among these, the novel superlubricious surface, slippery lubricant-infused porous surfaces (SLIPS), has garnered substantial attention in the antifouling field. The smooth interface formed by infusing a thin lubricant layer exhibits near-zero friction, long-term stability under hydraulic pressure, and robust repellency against both polar and nonpolar liquids. Its antifouling mechanism relies on the physical barrier of the lubricant layer rather than low surface chemical energy [143]. Molecular dynamics (MD) simulations and density functional theory (DFT) calculations reveal that, at the atomic scale, the non-polarity of the lubricant enhances the diffusivity of interfacial water molecules, leading to sustained nanoscale fluctuations at the liquid-liquid interface. These fluctuations are driven by the trade-off between enthalpy and entropy in minimizing the Gibbs free energy of the system. The absence of stable adhesion sites at the interface thereby inhibits the attachment of adhesion-related proteins [144].

However, conventional SLIPS are hindered by practical application limitations, such as lubricant depletion and poor controllability. Inspired by the self-defense mechanism of the skin in poison dart frogs, researchers incorporated ZIF-8 porous liquid (PL) as a lubricant into a silicone polyurethane (SPU) matrix. Porous liquids are fluid materials with permanent porosity, consisting of porous substances stably dispersed in sterically hindered fluids, and thus integrating continuous fluidity and inherent porosity. This bioinspired lubricious coating features a responsive antifouling agent release switch that enables reversible switching between “defensive” and “aggressive” antifouling modes. Such on-demand switching of the resulting SPIPS enhances antifouling efficiency, minimizes antifouling agent waste, and effectively mitigates the attenuation of antifouling performance induced by lubricant loss [145]. In another study, the shark-repellent property of the toxic mucus secreted by *P. pavoninus* (peacock sole) was mimicked. By introducing reversible chemical bonds between the lubricant and the matrix, and triggering lubricant release via photoexcitation, precise tunability of lubricant release kinetics was achieved [146]. When porous samples are fully swollen in water or polymeric lubricants (e.g., hyaluronic acid, sodium alginate), the expansion of the dissociation layer during dynamic swelling induces pore constriction, forming a semi-closed “gland-like pocket” structure that enables efficient entrapment and preservation of the lubricant. Notably, a mere 5 μL of lubricant suffices to sustain a superlubricious state for 3700 cycles in air [147]. Nevertheless, for electrodes with miniature size and intricate geometry—particularly microelectrode arrays—it remains extremely challenging to uniformly and reliably fabricate the required micro/nanostructures on their delicate architectures, as well as to achieve precise lubricant infusion. To address this issue, researchers have proposed a hybrid strategy combining surface laser ablation and surface-initiated polymerization to prepare patterned, durable hydrogel coatings. In contrast to conventional dip-coating and spray-coating methods, this approach enables spatial control over the hydrogel coating topography, thereby enabling application-specific customization. The incorporation of LA liposomes as lubricants not only endows the hydrogel coating with an ultra-low friction coefficient (∼0.002) but also significantly reduces reactive oxygen species (ROS) levels in the peri-wound tissue of mice, effectively suppressing scar formation and accelerating wound healing [119].

Graded regulation of coating surface properties also enables the construction of low-surface-energy “viscous layer” lubricious surfaces. Inspired by the super-hydrophilic “dentate layer” and low surface energy “mucus layer” structures of shark skin, researchers have proposed a facile strategy for the layer-by-layer fabrication of a triple-defensive antifouling coating. This coating comprises a hydrophilic mineralized catalytic nanoparticle (NP) layer and an overlying layer of hydrophobic perfluoro silane domains (F@NPs), which synergistically optimize antifouling, anti-adhesive, and anti-degradation performances [148]. The microscale amphiphilic surface formed by hydrophilic/hydrophobic domains generates nanoscale heterogeneous regions that can interfere with microbial recognition and adhesion, and significantly reduce the coefficient of friction (from 0.52 to 0.08) [149]. The implantation friction coefficient of the probe modified with the lubricious layer was reduced by 86%. Moreover, in the microglia and astrocytes adjacent to the probe, the expression levels of ionized calcium-binding adaptor molecule 1 (Iba1) and glial fibrillary acidic protein (GFAP) were significantly downregulated [108].

Furthermore, the core characteristic of achieving super lubricity lies in its coefficient of friction (COF) threshold (COF ≤0.01), which enhances the efficiency of mechanical systems by reducing frictional energy consumption. A semi-interpenetrating poly(3-sulfonylpropyl methacrylate) (PSPMK) lubricating network, constructed via a cartilage-mimetic semi-interpenetrating hydrogel interface, further reduces surface shear stress via the hydration lubrication mechanism. It slows down the propagation of frictional crack tips and induces a crack blunting effect. Under a high contact pressure of 2.8 MPa in PBS, it maintains stable super-lubricating performance even after 100,000 sliding cycles, with a COF as low as 0.0084 [150]. When combined with silicon nitride (Si_3_N_4_) and polytetrafluoroethylene (PTFE), semi-solid sub-nanometer nanowire (SNW) super lubricating materials exhibit an ultra-low COF (0.008–0.009), a short running-in period (≈39 s), and long-term frictional stability (12 h, >12000 cycles). Their superlubricity originates from the synergistic effects of the shear-thinning properties of SNWs, the adsorption film at the interface, and hydrodynamic effects [151]. In addition, some carbon dot/ionic liquid analog (CDs/ILAs) materials exhibit lubricating properties. Among them, CDs act as competing ligands to regulate the distribution of ILA cluster size, endowing them with both superlubricity and an ultra-high load-bearing capacity of up to 705 MPa. The competition between oxygen-containing functional groups on CDs and original hydrogen bond donors results in non-equal-sized clusters at different scales, increasing the probability of asymmetric contact and reducing interfacial shear resistance, thereby achieving lubrication dominated by the hydrodynamic mechanism [152].

Inspired by the insect-trapping mechanism of pitcher plants, researchers have developed a lubricated, immune-stealthy probe surface (LIPS) fully integrated with the probe. This surface possesses near-frictionless and anti-biofouling properties, enabling maximal in vivo electrode performance. LIPS can repel various bodily fluids, including artificial cerebrospinal fluid (aCSF), simulated body fluid (SBF), and blood, while maintaining excellent anti-biofouling capability. In vitro experiments confirmed that the adhesion rate of LIPS to plasma proteins and glial cells is less than 2%. Furthermore, LIPS reduced friction-induced pulses at the probe-tissue interface by 86%. Compared with bare probes, the signal-to-noise ratio (SNR) increased by 9-fold. By alleviating reactive gliosis around the probe, the signal measurement period could be extended to 16 weeks [153]. This lubricated, immune-stealthy LIPS can maximize the service life of implantable BCIs (Fig. 6D). However, there are currently few application examples of lubricating coatings in the design and research of anti-fouling brain–computer interface (BCI) electrodes. This is primarily attributed to the multiple challenges encountered by lubricating coatings in BCI electrode applications, which essentially represent the concentrated manifestation of a multi-field coupling problem involving biological, mechanical, and electrochemical processes: lubrication requires an ultra-low coefficient of friction and the maintenance of a continuous fluid interface, whereas BCI electrodes demand a long-term stable electrochemical interface to achieve high-SNR neural signal recording. The biological fluid environment can erode or contaminate the lubricating layer, leading to a significant increase in electrochemical impedance [92,154]. Additionally, lubricating coatings are typically insulating or weakly conductive, which conflicts with the charge-injection density requirements of BCI electrodes (>0.1 mC cm^−2^, up to 2.25 mC cm^−2^) [155]. Although conductive polymer modification can improve electrical conductivity (e.g., PEDOT:PSS achieves 60–600 S cm^−1^) [156,157], (R3.33) lubricating performance is drastically diminished. Moreover, current lubrication research focuses on dry friction or oil-lubricated environments [154], whereas brain tissue is a viscoelastic medium, and there are no reliable testing standards for its frictional dynamics at shear rates relevant to cerebrospinal fluid. Therefore, the application of lubrication technology in BCI electrodes necessitates the reconstruction of material design paradigms to address these complex challenges.

#### 3.1.3 Dynamic polymer brush structure

Polymer brush-modified interfaces, via the construction of liquid-like surfaces (LLS), have emerged as an effective anti-fouling strategy [158,159]. The flexible chain structure of molecular brushes provides a surface with a low coefficient of friction, which can reduce mechanical friction between electrodes and tissues, minimize tissue damage [160], and inhibit the adhesion and proliferation of fibroblasts as well as the adhesion and activation of platelets, thereby preventing thrombosis [122]. Liquid-like surfaces generally refer to coatings formed by covalently grafting highly flexible and dynamic polymer molecular brushes or alkyl monolayers onto solid substrates [161]. They exhibit liquid-like behavior and extremely low contact angle hysteresis (CAH), enabling liquids with different surface tensions (such as organic solvents and aqueous solutions) to slide off the surface easily. This liquid repellency does not depend on micro- or nanostructures, thereby avoiding the manufacturing difficulties of complex structures and their susceptibility to wear. In addition, polymer molecular chains possess high mobility in air, endowing them with fluid-like dynamic properties and overcoming the stability issues of lubricant-infused porous surfaces (SLIPS) caused by lubricant depletion [162]. Liquid-like surfaces can be achieved through various surface grafting techniques, including photo-initiated polymerization [163], atom transfer radical polymerization (ATRP) [164], controlled surface-initiated organocatalytic ring-opening polymerization [165], and hydrogen atom transfer reversible addition–fragmentation chain transfer (HAT-RAFT) polymerization [109] (Fig. 6E), thus enabling the immobilization of polymer brushes on surfaces with various curvatures. For example, when chondroitin sulfate (CS) polymer brushes are immobilized on plasma-treated silicon wafer substrates, the anti-adhesion to non-specific proteins in the early stage of implantation can further suppress acute inflammatory responses, and maintain anti-adhesion to FITC-albumin even after 4 weeks. In addition, the CS-coated group showed multiple pieces of evidence of improved blood–brain barrier (BBB) integrity: the intensity of IgG deposition was significantly reduced (indicating alleviated BBB leakage), weakened microglial activation might indirectly mitigate BBB damage, and the anti-fouling properties of the coating further blocked the surface adsorption of leaked IgG proteins. Ultimately, the impedance of neural electrodes decreased after coating, and no significant difference was observed between day 1 and day 7 [166].

The anti-fouling performance of polymer brushes is influenced by multiple factors. First, chemical composition is crucial. For instance, hydrophilic poly(2-ethyl-2-oxo-1,3,2-dioxaphospholane) brushes outperform polyester brushes in resisting various protein and bacterial fouling [165]; amphiphilic polymer brushes can couple the fouling-release capability of hydrophobic segments with the anti-adsorption performance of hydrophilic segments, synergistically enhancing anti-fouling properties and establishing an effective underwater oil-repellent barrier [167,168]. Second, surface grafting density significantly impacts anti-fouling performance. In high-density self-lubricating omniphobic covalently attached liquids (SOCALs) coatings, higher grafting density can reduce the contact area between liquids and the substrate, thereby decreasing adhesion [169]. Polymer brush systems based on dynamic covalent diketoenamine linkages can regulate grafting density by adding small molecules, overcoming the irreversibility limitation of traditional covalent grafting [170]. Third, molecular weight is also a key factor. Polymer chains with higher molecular weights generally exhibit better flexibility and fluidity, which can further reduce contact angle hysteresis (CAH) [171]. Molecular dynamics (MD) simulations have shown that zwitterionic brushes (pCBMA, pSBMA, pMPC) with longer flexible side chains tend to form larger unit cell sizes. The degree of surface hydration (pCBMA > pMPC > pSBMA > PEG) is consistent with the magnitude of the repulsive force exerted on lysozyme, revealing a positive correlation between surface hydration and atomic-scale anti-fouling performance. This is attributed to the formation of an interfacial dipole network within the first hydration shell, which acts to minimize surface dipoles and charges, thereby enhancing surface hydration and potentially suppressing electrostatic/dipole-induced protein adsorption [172].

Finally, the degree of polymerization also affects anti-fouling efficacy. Anti-fouling polymer brushes prepared via surface-initiated photoinduced electron/energy transfer (PET)-RAFT polymerization exhibit enhanced hydrophilicity, reduced stiffness, and a thickened hydration layer with increasing chain length. Consequently, protein adsorption capacity decreases significantly as the degree of polymerization increases [169]. Polymer brushes such as poly-sulfobetaine vinyl imidazole (PSBVI) [164] and polyethylene glycol (PEG) are widely utilized due to their excellent water solubility, anti-fouling properties, and biocompatibility. PEG-modified glycoproteins enhance hydrophilicity and stability, helping mask substrate defects and providing a smoother, more dynamic surface [173]. The anti-fouling mechanism of long-chain PEG-modified interfaces primarily stems from steric repulsion: when proteins approach, the polymer chains are compressed, decreasing in conformational entropy and increasing in free energy. The entropic effect drives chain expansion to repel contaminants [174], thereby reducing adhesion and activation of pro-inflammatory cells, as well as preventing the formation of fibrotic scar tissue capsules [134]. Bacterial adsorption tests also demonstrated that the thickness of the grafted polymer significantly reduces bacterial adhesion, and the adhesion amount decreases with increasing polymer brush thickness [164,175], particularly when hydrophilic brushes are used against *S. aureus* [165]. In addition, covalent immobilization of mucin brushes—derived from the mammalian innate immune system—onto vibrating microcolumns reduces the water contact angle of the polydimethylsiloxane (PDMS) substrate from 113° to 35°, thereby transforming the PDMS from hydrophobic to hydrophilic. This modification also significantly reduces the adhesion rate of *Pseudomonas aeruginosa* and facilitates biofilm removal (Fig. 6F) [110]. Inspired by mussels, the incorporation of catechol moieties into the structure of PEG block copolymers enables the construction of highly adhesive and highly anti-fouling coatings. Studies have shown that surface topology significantly influences anti-fouling performance: PEG coatings with a loop structure effectively reduce protein adsorption and are easy to clean, whereas PEG coatings with a brush structure tend to trap bovine serum albumin (BSA) between molecular chains, causing persistent fouling. Catechol-functionalized PEG block copolymers can be coated on gold surfaces; the protein adsorption capacity of their brush-like structure is approximately 8-fold lower than that of bare gold surfaces, and that of the loop structure is nearly zero [176].

Although liquid-like molecular brush coatings exhibit great potential in anti-fouling applications, their research still faces numerous challenges. Currently, it remains challenging to efficiently and facilely achieve functionalized polymer molecular brushes and translate their anti-fouling performance to target surfaces. Especially in complex biological environments such as the brain, research on the durability, biocompatibility, and mechanical strength of molecular brush coatings remains limited. In addition, developing synthetic methods that can precisely control the grafting density and structure of molecular brushes to optimize their liquid-like properties is a key direction that needs to be broken through in future research [167].

Integrating three types of anti-fouling strategies, their cross-domain transfer feasibility and intracranial scene adaptability are as follows: (i) zwitterionic coatings have achieved clinical translation in cardiovascular implants, with the highest broad-spectrum anti-fouling efficiency and the most extensive research. They are the most transferable universal solution for brain–computer interface electrodes. The ionic environment of cerebrospinal fluid may partially shield the charge of zwitterions, but usually does not affect their anti-fouling function. However, excessive crosslinking can increase the impedance of the electrode interface, so it is more suitable for low-current cortical recording electrodes; (ii) anti-adhesion lubricating coatings have been widely applied in minimally invasive interventional devices, which can simultaneously reduce implant friction damage. They are a complementary solution for preventing and controlling implant damage. The core limitation is that the lubricating layer is prone to loss and has poor long-term stability in the intracranial physiological environment. Moreover, most of them are insulating and incompatible with stimulation electrodes, making them suitable for short-term minimally invasive diagnostic electrodes; (iii) dynamic polymer brushes have been widely used in microfluidic devices, with minimal impact on the electrochemical performance of electrodes. They are a suitable solution for long-term high-density deep-brain recording electrodes. The core limitation is that the preparation process is complex and difficult to achieve a large-scale, uniform coating on brain-computer interface micro-nano electrode array.

### 3.2 Antibacterial coating

Antimicrobial coatings serve as the ultimate line of defense against biofilm formation. They eliminate adhered bacteria through chemical or physical means, preventing biofilm maturation and implant-related intracranial infections.

**Fig. 7:**
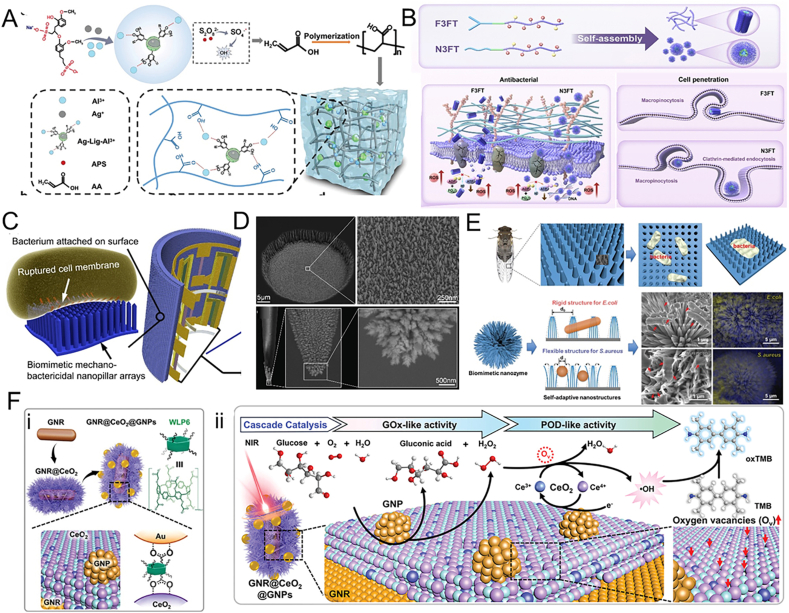
(A) PALA Hydrogel Design. Strategy: Self-adhesive, rigid, antibacterial, and conductive hydrogel gelation triggered by an Ag-Lig-Al^3+^ dynamic redox system. Reproduced with permission [181]. Copyright 2025, Wiley VCH. (B) Self-Assembled Dual-Function Nanopeptides. Mechanism: F3FT (nanorods) and N3FT (nanoparticles) self-assemble to penetrate cells, combating intracellular infections by eliminating bacteria and reducing inflammation. Reproduced with permission [182]. Copyright 2025, American Association for the Advancement of Science. (C) Orthopedic Implant Coatings. Design: Schematic of a dual-functional smart-coating foil engineered for orthopedic applications. Reproduced with permission [183]. Copyright 2023, American Association for the Advancement of Science. (D) NanoPt Coating Compatibility. Application: DC-deposited NanoPt coatings on both planar electrodes and conical probes, demonstrating versatility across different substrate geometries. Reproduced with permission [155]. Copyright 2020, American Chemical Society. (E) Bioinspired Bacterial Capture Structures. i. Natural Template: Cicada wing nanocone arrays showing natural bacterial capture and bactericidal properties. ii. Synthetic Nanozyme: SEM and schematics of NiCo_2_O_4_ nanozymes that self-adapt their hierarchical structure to capture various bacterial morphotypes (red arrows) [184]. Copyright 2024, Wiley VCH. (F) Bio-Cocklebur Catalytic Platforms. i. Structure: Preparation and structural overview of GNR@CeO_2_@GNPs “bio-cockleburs.” ii. Activity: Demonstration of plasmonic-enhanced cascade catalysis in vitro using the bio-cocklebur platform. Reproduced with permission [185]. Copyright 2024, Wiley VCH.

The implantation surgery of brain-computer interface (BCI) electrodes carries major risks of infection, hemorrhage, and stroke [8]. Among these, infection is a critical issue encountered during the early post-implantation phase and is closely associated with exposure to exogenous substances. Even months to years after implantation, delayed bacterial infections may still occur, or infections from other parts of the body may spread to the brain via the bloodstream, and tissue damage caused by electrode wear further increases the infection risk [177,178]. Bacterial infections are associated with exacerbation of inflammatory response in the wound area; therefore, antibacterial requirements at the implantation site are crucial throughout the entire lifespan of the electrode. Invading bacteria adhere rapidly to the surface of bioactive implants and form biofilms to survive in unfavorable host environments. The polymeric matrix of biofilms impedes the penetration of antibiotics and immune cells, rendering traditional antibiotic regimens ineffective and leading to persistent and recurrent infections. Studies have shown that bacterial adhesion is the primary cause of biofilm formation [179]. These infections not only correlate with intracranial infections, permanent neural damage, and deterioration of electrode signal quality but may even threaten the patient's life. Direct bacterial killing represents another major approach distinct from the aforementioned anti-adhesion antibacterial strategies. Loading of antibacterial agents—such as tetracycline (TC) and dexamethasone (DEX)—in combination with bacterial cellulose enables sustained release from interface electrodes, correlating with reduced the growth of Gram-negative and Gram-positive bacteria and alleviated inflammatory responses [55]. In addition, various nanoparticle composites have demonstrated antibacterial potential, including Cu-polyurethane/MXene elastomers [180], PALA bimetallic phenolic network hydrogels co-constructed by Al^3+^ and Ag-Lignin NPs (Fig. 7A) [181], ZnO nanoparticle-anchored microfibers [186], silver nanowires (AgNWs) [187], and methacrylate alginate (MAA) hydrogel electrode patches. The latter can mediate immune responses and prevent bacterial infections in the microenvironment at the contact interface [188]. Notably, electrodes composed of N-acryloyl glycinamide (NAGA) and hydroxypropyltrimethyl ammonium chloride chitosan (HACC) exhibit inhibition rates exceeding 80% (10 h) against *E. coli* and 95% (7.5 h) against *Staphylococcus epidermidis*, respectively [189], demonstrating excellent antibacterial performance.

#### 3.2.1 Loaded antimicrobial peptides

Compared with traditional antibiotics, quaternary ammonium compounds (QACs), and surface modification strategies involving metals and their oxides, antimicrobial peptides (AMPs)—important components of the innate immune system of organisms—possess unique mechanisms of action and distinct advantages. AMPs can specifically target and bind to the cytoplasmic membrane of pathogenic bacteria, inducing membrane protein dislocation, inhibiting cell wall synthesis and cellular respiration, and ultimately eradicating pathogenic bacteria [190]. In anti-infective therapy, AMPs are regarded as highly promising next-generation antibacterial agents due to their relatively low risk of inducing bacterial drug-resistant mutations and generating cytotoxicity. However, technical challenges remain in the safe and effective integration of AMPs onto the surfaces of electrode implants and in achieving precise release.

There are diverse approaches to integrating AMPs with implants for achieving efficient loading and precise release. These strategies include directly loading AMPs into stimulus-responsive electroactive hydrogels within integrated electronic implant systems, with drug release controlled via voltage-modulated electrodes [191]; or directly coating and immobilizing AMPs on the implant surface [192,193]. In addition, material surface modification is also a crucial approach. For instance, by leveraging the strong adhesive property of 3,4-dihydroxy-L-phenylalanine (DOPA)—abundant in mussel foot proteins—and combining it with NHS/EDC-activated peptide modification technology, fusion peptide-engineered polyetheretherketone (PEEK) implants have been successfully fabricated, endowing them with triple functionalities: antibacterial activity, osteogenesis, and angiogenesis promotion [194]. DOPA derivatives rich in click chemistry azide termini can integrate anti-infectivity and osteoinductivity into PEEK implants. Their “clickable” feature overcomes the drawbacks of classical dopamine surface chemistry, which may consume biomolecular active sites and thereby impair functionality [190]. For example, the poly(Phe10-stat-Lys12)-DOPA antibacterial coating with DOPA end groups exhibits high-efficiency antibacterial activity and inhibits the expression of drug-resistant genes [195]. Furthermore, AMPs are bonded to DOPA via a “linker” composed of a specific amino acid sequence, which can be precisely cleaved by wound-specific enzymes (e.g., human neutrophil elastase, hNE) to achieve targeted release of AMPs. Experiments have shown that the MP−(hNE)3−[Nle5]-SHAP1 complex effectively inhibits bacterial growth only after hNE cleaves the linker to release the SHAP1 antimicrobial peptide, and it is almost inactive in the absence of hNE. This provides a novel approach for precisely controlling AMP release [196]. In addition, AMPs can be firmly bonded to the surface of silver nanoparticles (AgNPs) via hydrogen bonds, forming complexes. Particularly, AMPs with terminal amino groups exhibit higher synergistic efficiency with AgNPs, as their complexes can promote the production of reactive oxygen species (ROS). Meanwhile, the cell spreading area on Ag@AP/SF is larger than that on Ag/SF, indicating that AMPs effectively reduce the cytotoxicity of AgNPs and improve biocompatibility [197].

The application of AMPs in brain-computer interfaces (BCIs) still faces challenges, primarily due to their poor stability in physiological environments, which severely hinders their translation into clinical applications. The antibacterial activity of many AMPs is significantly reduced or even abrogated at serum or physiological salt concentrations, due to their binding to serum anionic proteins and susceptibility to protease hydrolysis. Therefore, future research directions should focus on multifunctional self-assembled peptide-based nano-systems with appropriate hydrophobicity and responsiveness (Fig. 7B) to overcome these stability barriers. This will effectively enhance the performance and durability of AMPs in complex physiological environments, thereby providing novel strategies for the long-term stable service of BCIs [182].

#### 3.2.2 Micro-nano sterilization structure

Although drug loading is the mainstream and most effective antibacterial strategy, for interface electrodes targeting long-term antibacterial performance, the therapeutic efficacy of drugs tends to diminish over time as active ingredients are depleted. Worse still, this approach may even induce bacterial resistance, thereby posing new challenges to the prevention and treatment of implant-associated infections. In contrast, micro-/nanostructure-mediated sterilization offers a safe and sustainable anti-fouling solution. First, nanostructured surfaces can significantly mitigate microbial adhesion. The fundamental mechanism underlying this effect is that nanostructures force microorganisms into point contact rather than surface contact with the substrate, thereby drastically reducing both the adhesion area and the adhesive strength [116]. Second, the mechanical interactions between micro/nanostructured surfaces and bacteria can be harnessed to kill bacteria or to inhibit their attachment and subsequent biofilm formation, rendering this strategy a research hotspot in the fields of anti-fouling and antibacterial research. Therefore, physically bactericidal micro/nanostructures are promising as a pivotal strategy for preventing implant infections, particularly by impeding initial bacteria adhesion. For instance, specific polymeric nanocolumn arrays not only exhibit broad-spectrum and highly efficient antibacterial activity but also demonstrate excellent biocompatibility, as they show no cytotoxicity toward mammalian cell lines derived from bone, skin, and muscle tissues after 48 h of incubation [183] (Fig. 7C). Mechanisms of mechanical sterilization are primarily categorized into two types (1) nanoarray structures (e.g., nanocolumns with varied morphologies and dimensions) that induce tensile rupture of bacterial cell membranes; and (2) sharp nano-edges (e.g., graphene nanosheets and MXenes) that physically disrupt bacterial cell membranes via a “nano-knife” effect [198,199]. A diverse range of fabrication techniques is available for preparing micro/nanostructures, including deposition methods [155,185,200](Fig. 7D), template-assisted synthesis [201], etching processes [183,202], in situ growth [203], spray coating [204], and self-assembly [205]. Additionally, bioinspired antibacterial nanomaterials—such as the hierarchical nanostructures on dragonfly and cicada wings, which disrupt bacterial membranes through physical and mechanical forces—provide a novel paradigm for the design of next-generation antibacterial materials [183,202].

Nanozymes, a category of nanomaterials with enzyme-mimicking properties, have been extensively reported for antibacterial applications, as they disrupt bacterial membranes and induce bacterial apoptosis by mimicking the catalytic generation of reactive oxygen species (ROS) [206]. For instance, the Bi-doped Bi-PCN-222 metal–organic framework (MOF) nanozyme can produce abundant ROS to eradicate bacteria via Schottky junction-driven charge transfer as well as intrinsic oxidase- and peroxidase-like activities. Furthermore, when Bi-PCN-222 interacts with bacteria in physiological microenvironments, its optimal redox potential can trigger electron transfer across and within bacterial membranes [207], thereby interfering with cellular respiration, inducing oxidative stress, and damaging bacterial DNA and cell membranes [204]. However, despite the advantages of high stability, low cost, recyclability, and multifunctionality, nanozymes still exhibit inferior antibacterial activity compared with conventional antibiotics. To address this limitation, researchers have engineered novel nanozymes that integrate mechanical disruption and catalytic activity via bioinspired strategies, such as mimicking the surface architectures of cicada and dragonfly wings. For example, the NiCo_2_O_4_ nanozyme, which combines the mechanical rupture capability of its surface nanocone topography with its peroxidase-like catalytic oxidation function, can capture bacteria of diverse morphotypes [205]. The driving force for such capture and destruction originates from the physicochemical adhesion between nanozymes and bacterial cells; membrane rupture occurs when the localized membrane tension exceeds the tensile strength threshold of the bacterial cell envelope [208]. Against different bacterial strains, rigid nanometallic nanostructures can pierce bacteria with large volumes and thin cell walls, whereas flexible nanofiber architectures efficiently entrap small-sized bacteria with thick cell walls [184] (Fig. 7E).

Furthermore, a biomimetic “Xanthium"-inspired nanozyme material (GNR@CeO_2_@GNPs) integrated with photothermal properties is fabricated by depositing spiky CeO_2_ nanostructures and GNPs onto gold nanorods (GNRs) [185]. The spiky architectures can penetrate bacterial cell membranes via a “mechanical invasion” mechanism, thereby anchoring the bacteria firmly in place. Upon near-infrared (NIR) laser irradiation, the synergistic effects of photothermal conversion and catalytic activity [205] rapidly elevate the local temperature, disrupting bacterial cell walls while simultaneously generating highly reactive oxygen radicals (e.g., ·OH). These effects dissipate membrane potential and downregulate ATP levels, ultimately enabling efficient thermophoresis-driven targeted eradication of multidrug-resistant bacteria (e.g., MRSA) and alleviation of inflammation (Fig. 7F) [185,209] [210]. However, under conditions of severe bacterial contamination, dead bacteria and cellular debris tend to accumulate on rough surfaces, which impairs subsequent bactericidal efficacy. To resolve this issue, researchers replicated cicada wing-mimetic polycarbonate (PC) nanopores via hot-press molding, followed by polydopamine (PDA) modification to reduce surface hydrophobicity. Subsequently, ZIF-8 nanoparticles doped with 3,3′-diaminodipropylamine (DADP) were grown in situ on the pore surfaces. This composite structure achieves synergistic antibacterial activity through the mechanical bactericidal action of nanopores and the pH-responsive release of DADP [163]. It effectively eliminates adherent bacteria under physiological conditions and triggers the on-demand release of bactericidal agents in response to bacterial proliferation during severe contamination. Precisely this efficient bacterial clearance and inhibition create a critical prerequisite for the PDA-modified nanopores—exposed after ZIF-8 degradation—to exert their biological functions safely: the modified surface can selectively pierce residual bacteria while significantly enhancing the adhesion and proliferation of fibroblasts [201].

In the design of biointegrated intelligent electrodes, integrating bioinspired micro/nanostructures and sensing functionalities onto flexible polymer substrates offers immense application potential. For instance, a multichannel strain-sensing array is constructed using single-crystalline silicon nanomembranes and fabricated via transfer printing, with a bioinspired mechano-bactericidal nanocolumn array—featuring precisely tunable geometric parameters (pitch/diameter/height)—integrated onto its surface. This array is fabricated by a hybrid nanofabrication technique, following a specific workflow: (1) fabricating wafer-scale colloidal masks using monodisperse polystyrene nanospheres; (2) forming porous templates through oxygen plasma etching and metal deposition; (3) preparing vertical nanopores via deep silicon reactive ion etching; and (4) depositing polyamic acid precursors followed by thermal crosslinking to yield flexible crosslinked polyimide films. Functional validation experiments indicate that this coating can effectively eliminate intradermal bacterial colonies, reducing the counts of *Staphylococcus aureus* and *Pseudomonas aeruginosa* by several orders of magnitude, while concurrently mitigating neutrophil infiltration and tissue damage. Moreover, the integrated system maintains structural and functional stability in biological fluid environments. The integrated strain-sensing array achieves a strain measurement resolution of 0.01% at a low operating voltage of 1–5 V, with a spatial resolution that is 80-fold superior to that of conventional flexible metal foil strain gauges [183].

However, implementing nanoscale functionalities imposes stringent requirements for fabrication precision and process control, pushing the boundaries of engineering and materials science. Core challenges include the following aspects: (i) Scalability of high-resolution, high-fidelity nanofabrication: Electron beam lithography offers ultrahigh precision but is not amenable to mass production; conversely, nanoimprint lithography enables high-throughput replication yet suffers from high mold costs and poor mold durability; (ii) Guarantee of large-area uniformity and structural consistency: Structural irregularities or defects can compromise antibacterial performance and even facilitate bacterial colonization; (iii) In vivo durability: Long-term mechanical abrasion caused by surrounding tissues can degrade nanostructures, thereby impairing their long-term functionality.

In addition, the long-term stability of bioinspired multifunctional surfaces remains a critical unmet need. The mechanical strength of immobilized nanostructures is often insufficient to withstand the physiological loads encountered during implantation. Potential nanostructure detachment not only results in functional failure but may also trigger adverse immune responses. Therefore, developing more robust materials, scalable fabrication processes, and conducting in-depth in vivo stability evaluations are the key prerequisites for unlocking the full potential of this technology and facilitating its clinical translation [211].

Considering the characteristics of the two types of antibacterial strategies, sterilization strategies are the most feasible for cross-field transfer. Among them, antimicrobial peptides have been clinically applied in orthopedic and ophthalmic implant devices, with a broad bactericidal spectrum, rapid onset of action, and low systemic toxicity. They are the preferred solution for short-term infection prevention and control during the perioperative period of brain–computer interfaces. The core limitation is that they are easily hydrolyzed and inactivated by proteases in the intracranial environment, posing a risk of inducing bacterial resistance, therefore making them suitable for short-term implantable surgical electrodes. Moreover, micro-nano structural physical sterilization has been applied in interventional devices, with no risk of resistance and a long-lasting antibacterial effect. It is the most feasible approach for permanent antibacterial treatment of long-term-implanted electrodes. The core limitation is that increased surface roughness can exacerbate protein adhesion, contradicting the core goal of anti-fouling, and the structure is prone to wear and detachment, posing a risk of nanomaterial neurotoxicity. It is suitable for rigid stimulation electrodes with long-term permanent implantation.

The cross-field transfer of anti-fouling and antibacterial technologies is a core, feasible path to address gaps in infection prevention and control for brain-computer interface electrodes and reduce the risk of clinical translation. The mature application of related technologies in implantable devices such as cardiovascular stents and cochlear implants provides a preliminary scientific foundation for the design optimization of brain-computer interface electrodes. However, there are still two deficiencies in this field: (i) a lack of exclusive adaptation optimization for intracranial scenarios. Most studies focus on direct cross-field transfer without fully matching the core constraints of intracranial implantation. Specifically, there is zero tolerance for neurotoxicity, potential stability and activity attenuation of antibacterial substances due to the special physicochemical environment of cerebrospinal fluid, stringent requirements for compatibility with electrode electrochemical performance, and the high risks of disability and death from intracranial infection. (ii) The combination of anti-fouling and antibacterial functions violates the temporal sequence of biofilm formation. Almost all existing studies simply superimpose and synchronize the two functions, ignoring that the anti-fouling function should act in the hyperacute phase (blocking initial adhesion) and the antibacterial function should act in the late acute phase (killing colonizing bacteria). Synchronization not only fails to synergize but may also mutually weaken functions, which is an important reason many composite coatings are effective in vitro but fail in vivo over the long term. Thus, an intelligent triggering mechanism allows the coating to sense post-colonization cues and switch from anti-fouling to antibacterial at the right time, achieving spatiotemporal synergy.

## 4 Reduce the damage caused by long-term implantation

The cross-field transfer of anti-fouling and antibacterial technologies is a core, feasible path to address gaps in infection prevention and control for brain-computer interface electrodes and reduce the risk of clinical translation. The mature application of related technologies in implantable devices such as cardiovascular stents and cochlear implants provides a preliminary scientific foundation for the design optimization of brain-computer interface electrodes. However, there are still two deficiencies in this field: (i) a lack of exclusive adaptation optimization for intracranial scenarios. Most studies focus on direct cross-field transfer without fully matching the core constraints of intracranial implantation. Specifically, there is zero tolerance for neurotoxicity, potential stability and activity attenuation of antibacterial substances due to the special physicochemical environment of cerebrospinal fluid, stringent requirements for compatibility with electrode electrochemical performance, and the high risks of disability and death from intracranial infection. (ii) The combination of anti-fouling and antibacterial functions violates the temporal sequence of biofilm formation. Almost all existing studies simply superimpose and synchronize the two functions, ignoring that the anti-fouling function should act in the hyperacute phase (blocking initial adhesion) and the antibacterial function should act in the late acute phase (killing colonizing bacteria). Synchronization not only fails to synergize but may also mutually weaken functions, which is an important reason many composite coatings are effective in vitro but fail in vivo over the long term.

### 4.1 Adaptive super flexibility

Adaptive ultra-flexible design is the core means to ensure the long-term stability of electrode implantation. By modifying materials and optimizing structures, it eliminates the mechanical mismatch between the electrode and brain tissue, thereby blocking the endogenous driving factors that repeatedly activate chronic inflammation from its root. This chapter describes the material strategies for the long-term structural failure phase, which is the final stability-guaranteeing link of the temporal stage-specific design paradigm. At the tissue level, chronic mechanical stimulation is a key factor causing persistent damage to neurons and glial cells, repeated activation of innate immune responses, and release of damage-associated molecular patterns (DAMPs). This promotes the activation of microglia or macrophages, the secretion of pro-inflammatory cytokines, and the progressive thickening of fibrotic capsules, which constitute the primary endogenous drivers of the persistence or recurrence of non-infectious chronic inflammation. In particular, the mechanical mismatch between rigid probes and brain tissue induces micromotion, directly damaging neural tissue and triggering immune responses. Although this type of inflammation exhibits similar manifestations to those induced by other etiologies, its core driving mechanism is the sustained action of physical forces. Therefore, achieving ultra-flexibility enables better buffering of intracranial and extracranial pressure fluctuations, reduces the risks of tissue damage, hemorrhage, and immune inflammation, and represents the most direct approach to resolving the mechanical mismatch conflict of implantable electronic devices for brain-computer interfaces (BCIs) [96,139,212]. The key lies in designing materials with modulus matching that of the surrounding tissue, thereby eliminating the mechanical mismatch, which serves as the fundamental source of micromotion.

As one of the softest and most fragile tissues in the human body, brain tissue is highly vulnerable—its neural axons rupture at a strain of approximately 18%, which means rigid implants inevitably damage neural tissue during implantation. Unlike other tissues in the body, brain tissue is protected by robust structures such as the dura mater; it is scarcely exposed to external mechanical stress and does not generate internal tension analogous to that in muscles or blood vessels. Therefore, brain tissue is exceptionally sensitive to exogenous mechanical stress and highly susceptible to injury. Furthermore, although brain tissue possesses certain regenerative mechanisms, self-repair following neural injury is extremely difficult. This is primarily attributed to the adverse microenvironment induced by post-traumatic inflammation, the absence of a healthy extracellular matrix, and the formation of glial scars, which impede neuronal survival, regeneration, and axonal outgrowth [213,214]. Flexible electrodes are recognized as a pivotal technology for implementing high-dimensional stimulation, with distinct advantages in tissue integration, biosafety, spatial coverage, electrode density, and long-term stability [103]. In terms of tissue integration and biosafety, flexible microelectrodes can conform tightly to neural tissue, reducing the stimulation threshold to as little as 1.5 μA (0.25 nC/phase). This value is far below the current intensities, ranging from hundreds of microamperes to milliamperes, required by rigid electrodes, thereby minimizing the risk of tissue damage. Immunohistochemical analyses demonstrated that, compared with the contralateral homotopic region of the brain, the electrode implantation area exhibited no significant neuronal loss and did not show marked upregulation of inflammatory markers, including astrocytes and microglia. These findings fully validate the long-term stability and biocompatibility of flexible electrodes [[215], [216], [217]].

#### 4.1.1 Ultra-flexible hydrogel

**Fig. 8:**
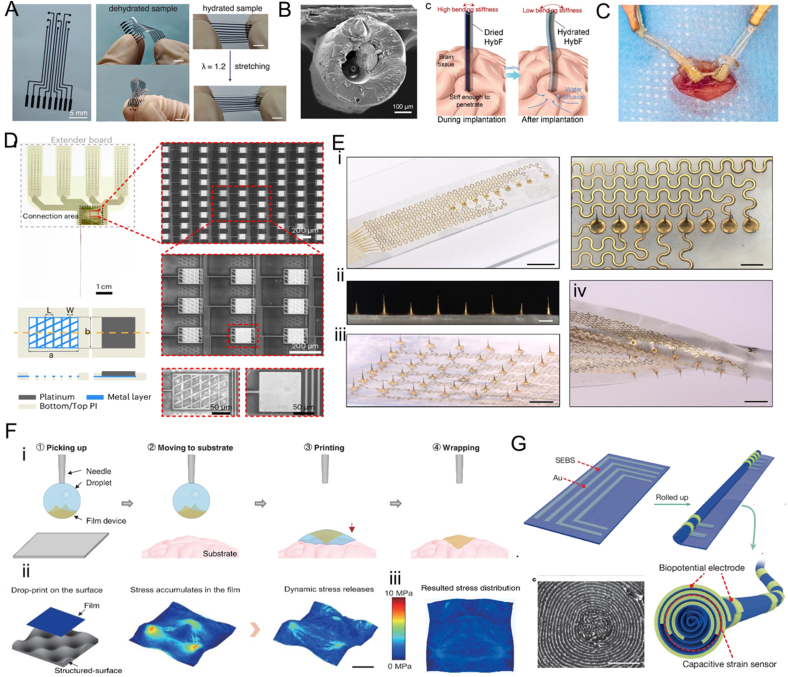
(A) Flexible Hydrogel Bioelectronics. Capability: Images of a 9-channel hydrogel device that is free-standing and flexible, capable of 1.2x stretching upon hydration. Reproduced with permission [218]. Copyright 2025, Wiley VCH. (B) Hybrid Probe Mechanics (HybF). Structure: SEM cross-section of the hybrid hydrogel probe. Mechanism: Conceptual illustration of adaptive stiffness; the dry AlgMX fiber is rigid enough for insertion but softens into a flexible state after absorbing water post-implantation. Reproduced with permission [12]. Copyright 2025, American Chemical Society. (C) Nerve Interface. Application: Surgical demonstration of a mouse sciatic nerve wrapped with 3D-printed hydrogel electrodes. Reproduced with permission [219]. Copyright 2023, Springer. (D) High-Density Neuroscroll Probe. Hardware: Photo and SEM of a 50-mm-long, 1024-channel Neuroscroll probe connected to an extender board via the FCB method. Architecture: Schematics showing the top and side views of the mesh I/O pad and target board interface. Reproduced with permission [220]. Copyright 2024, Springer. (E) Stretchable Microneedle Electrode Arrays (SMNEA). i. Design: Optical images of microneedle electrodes with serpentine interconnects on glass and silicone substrates. ii-iv. Versatility: Side and angled views showing varying needle lengths (800–1500 μm) and the array's durability under stretching and twisting. Reproduced with permission [17]. Copyright 2024, American Association for the Advancement of Science. (F) Drop-Printing vs. Conventional Transfer. i. Process: Schematic of the drop-printing method and the resulting dynamic stress release during deformation. ii-iii. Comparison: SEM evidence showing uniform stress distribution in drop-printed films versus stress concentration in soft stamp transfer methods. Reproduced with permission [221]. Copyright 2025, American Association for the Advancement of Science. (G) Earthworm-Inspired NeuroWorm. Biomimicry: Concept and fabrication process of the NeuroWorm, including an SEM cross-section showing its earthworm-inspired structural design. Reproduced with permission [26]. Copyright 2025, Springer.

Hydrogel-based electrodes can closely match the mechanical properties of brain tissue, and are associated with reduced inflammation and glial scar formation [218] (Fig. 8A), as well as enhancing neuronal integration. This favorable tissue–electrode interfacial integration is critical for improving the long-term signal recording performance of flexible microelectrode arrays [222]. From a mechanical standpoint, to achieve conformal contact with host tissue, electrode materials must recapitulate the unique mechanical attributes of brain tissue—including an elongation at break of 20% to 75% and modulus matching.

As soft biomaterials that mimic native biological tissues [223], hydrogels typically exhibit an elastic modulus ranging from 10 Pa to 100 kPa, making them ideal candidates for matching the mechanical properties of brain tissue (modulus: 0.1–1 kPa). Common ultra-flexible hydrogel materials include polyvinyl alcohol [11], acrylate-based polymers [224], polyethylene oxide (PEO) [225], and sodium alginate-based hydrogels (Fig. 8B) [12]. For instance, flexible scaffolds that recapitulate the neural tissue microenvironment were fabricated by incorporating carbon nanomaterials into an alginate hydrogel matrix, followed by freeze-drying. Neural progenitor cells seeded on these scaffolds formed neuronal networks spanning the three-dimensional (3D) material and differentiated into astrocytes and myelinated oligodendrocytes, thus offering a novel strategy for the functional reconstruction of neural interfaces [226].

Tensile performance is critical for flexible electrodes; therefore, developing hydrogel materials with ultra-stretchability and high mechanical strength represents a key research priority. This can be achieved via multiple approaches: polymer nanocrystalline structures provide a durable elastic matrix, while nano conductive fillers form an anisotropic conductive network through structural reconstruction and rearrangement, thus ensuring stable electrical conductivity under tensile deformation [224]. For instance, carbon nanotubes with high aspect ratios were incorporated into semi-crystalline polyvinyl alcohol (PVA) hydrogels, and anisotropic conductive pathways were constructed via cyclic stretching to optimize electrode performance. The resulting anisotropic conductive hydrogel electrodes (diameter: 187 ± 13 μm) retained excellent mechanical stability and high stretchability (64.5 ± 7.9%) even after 20,000 stretching cycles at 20% strain, while exhibiting low electrochemical impedance (33.20 ± 9.27 kΩ for a 1 cm^2^ electrode at 1 kHz). Experimental results revealed that the axial reconstruction and alignment of nanofillers induced an anisotropic reduction in impedance along the tensile direction. These hydrogel fibers maintained stable conductivity under long-term cyclic strain and successfully recorded spontaneous electrophysiological signals from motor neurons in the ventral horn of mouse spinal cords for up to 8 months, demonstrating suitability for neural interfaces with dynamic biological tissues [11]. In addition, the synergistic effects of hydrogen bonds and metal-ligand coordination bonds between polymer chains and nanoparticles act as “sacrificial bonds” that dissipate energy during stretching, thereby endowing hydrogels with superior mechanical stability [181]. Furthermore, hydrogel-based electrodes exhibit adaptive mechanical properties in response to environmental stimuli such as humidity, temperature [227], and mechanical stress-strain variations. The mechanical properties of bioelectrodes can be tuned by adjusting sodium alginate concentrations [228]. A composite structured electrode (HybF), consisting of a sodium alginate/MXene conductive hydrogel core and a PLGA/MgO insulating shell, achieves dry–wet stiffness switching based on the superhydrophilicity of polysaccharides and MXene: its bending stiffness reaches approximately 1 N/m in the dry state, which meets the mechanical support requirements for implantation; in the wet state, the bending stiffness decreases to 0.3 N/m, and the PLGA shell has a modulus on the kPa scale that closely matches that of brain tissue [12].

Poly(3,4-ethylenedioxythiophene):poly(styrenesulfonate) (PEDOT:PSS) has attracted considerable attention as a flexible electrode material owing to its tunable electrical properties, solution-processability, biocompatibility, and optical transparency. Via the flexible self-polymerizing network structure formed by a semiconductor polymer gel active layer and an ionic gel electrolyte [229], all-gel devices exhibit an elongation at break of up to 50% [13]. Studies have demonstrated that incorporating D-sorbitol into PEDOT:PSS solutions, followed by drying and annealing, induces the recrystallization of PEDOT-rich domains into interconnected and highly conductive nanofibers. Subsequent immersion of the resulting films in water or phosphate-buffered saline (PBS) triggers swelling of the hydrophilic PSS-rich matrix in the thickness direction, yielding soft and stable hydrogels. When the D-sorbitol concentration is increased from 1% to 9% (w/v), the tensile modulus increases linearly from 3.2 MPa to 7.3 MPa, whereas the maximum tensile strain decreases from 35% to 26%; the compressive modulus also rises from 0.7 MPa to 2.5 MPa. Wrapped around the sciatic nerves of freely moving rats, these electrodes significantly mitigate inflammation while preserving normal motor function [230]. The limitation of poor water solubility inherent to conventional polymeric semiconductors can be overcome via a solvent-exchange double-network strategy: polymeric semiconductors bearing hydrophilic side chains (p(g2T-T)) are mixed with hydrogel monomers (acrylic acid, AAc) in dimethyl sulfoxide (DMSO), followed by UV-crosslinking to form a gel. Subsequently, solvent exchange—whereby DMSO is replaced with water—induces rapid in-situ precipitation of the polymeric semiconductors to form a conductive network. This approach yields tissue-mimicking ultrashort mechanical properties and a high carrier mobility (1.4 cm^2^/V·s) [14].

The complex physiological microenvironment of the human body poses significant challenges to the long-term electrochemical stability of flexible electrodes, primarily manifested as electrical signal drift. To mitigate the increase in electrode resistance caused by tissue tensile deformation, researchers embedded a silver/liquid metal (SLM) layer within the hydrogel network. Leveraging the anchoring effect between eutectic gallium-indium (EGaIn) and silver (Ag) flakes, the SLM layer exhibited only a ∼10% increase in resistance under 30% tensile strain. Under conditions of prolonged use, cyclic stretching, and varying tissue adhesion states, the polyaspartic acid-modified dopamine/ethyl ionic liquid hydrogel (PDEH-SLM) patch electrodes maintained the capability to record high-fidelity electrophysiological signals, enabling the effective diagnosis of injuries to nerves, including the sural nerve, common peroneal nerve, median nerve, and radial nerve [15]. Furthermore, the body's humid physiological environment compromises the interfacial stability between conductive polymers and electrode substrates. Particularly during long-term electrical stimulation, PEDOT:PSS coatings undergo continuous volume expansion and contraction, leading to structural damage such as cracking and delamination. These structural defects, in turn, lead to a drastic decline in electrochemical performance, along with the formation of cerebral scars and inflammatory responses—limiting the clinical utility and reliability of such devices for neural diagnosis and therapy.

To address this issue, researchers prepared PEDOT:poly(styrenesulfonate-co-4-vinylpyridine) (Poly(SS-4VP)) interpenetrating network hydrogels via a three-step process: (1) chemical grafting of the functional long-chain polymer Poly(SS-4VP) onto metal substrates; (2) electrochemical deposition of conductive polymers (e.g., PEDOT); and (3) subsequent chemical crosslinking. Numerical simulations demonstrated that the synergistic effects of covalent anchoring of long-chain polymers and chemical crosslinking collectively enhance the long-term interfacial stability. This approach not only enables the formation of a robust conformal interface between conductive polymer hydrogels and rigid electrodes but also endows the composite electrodes with mechanical flexibility, high electrical conductivity, and long-term electrochemical stability. When bioelectrodes coated with PEDOT:Poly(SS-4VP) hydrogels were implanted into the hippocampus of mice, the conductive polymer hydrogel/electrode interface remained stable throughout a 4-week electrophysiological recording period. The impedance value at 1 kHz was consistently maintained below 250 kΩ, thus ensuring high-resolution electrophysiological signal acquisition [71].

The hydrogel electronic devices discussed above are typically composed of a hydrogel matrix and two components: metal circuits and electronic components, that is, hydrogel-encapsulated circuits. However, the core circuitry is still made of hard metal, and there is currently no method to efficiently construct customizable flexible circuits in a hydrogel matrix. To address this challenge, researchers have developed a 3D printing conductive ink based on microgel particles. This ink is based on calcium alginate and contains acrylamide monomers, crosslinking agents, and free radical initiators, with a particle size of approximately 20 μm. Firstly, solidifying calcium alginate and refining it into microgel particles can serve as the “ink” for 3D printing. After printing is complete, polyacrylamide is triggered by heating to finally shape the electronic device. Further, the microgel particles are mixed with a small amount of microsilver sheets and additives to produce conductive ink materials. This conductive hydrogel ink can freely construct flexible circuits with three-dimensional structures within the microgel particle matrix via embedded printing methods. Under an electron microscope, microsilver sheets are distributed like capillaries in the gaps between the microgel particles, forming a 3D soft network that enables the free flow of current. As the silver sheet is mainly confined to the interface of the hydrogel micro-particles, the electrical conductivity of this ink can be as high as 1.4 × 10^3^ S/cm. This fully hydrogel electrode that provides electrical stimulation can be wound around the sciatic nerve of mice through a simple surgical procedure. Under stimulation with a 1Hz pulse voltage, the 3D-printed electrodes can elicit regular, large-angle movements of the mouse's legs at a voltage as low as 100 mV [219] (Fig. 8C).

#### 4.1.2 Elastomers

Integrating electrode arrays onto stretchable substrates (e.g., polyimide, PI) is a conventional approach to fabricating flexible electrodes [231] (Fig. 8D). However, this configuration faces the critical challenge of interfacial stress concentration arising from the substantial mismatch in elastic moduli between the constituent materials, which, in turn, compromises device performance and long-term durability. Although PI itself has a relatively high modulus, ultra-thinning the material (e.g., to several micrometers) enables greater overall flexibility. Its superior pliability allows better adaptation to brain tissue micromotion and mitigates foreign body reactions; in human patients, the detection threshold for intracortical microstimulation (ICMS) remained relatively stable for over 1500 days. Moreover, PI exhibits exceptional durability; for instance, PI microelectrode arrays with a thickness of 10–15 μm can withstand 1 billion electrical pulses without degradation [232]. To further optimize performance, researchers modified the surface of flexible electrodes with reduced graphene oxide/poly(3,4-ethylenedioxythiophene):poly(styrenesulfonate) (rGO/PEDOT:PSS) composites. This modification improves interfacial compatibility with neural tissue, alleviates inflammatory responses, and enhances the signal-to-noise ratio (SNR) of neural signal recording [233]. Following successful implantation, the modified electrodes can detect neuronal firing correlated with motor states in mice, thereby providing a viable platform for investigating functional synergy across brain regions and the crosstalk between electrophysiological and electrochemical signals [32]. Inspired by the interlocking architecture between plant roots and soil, researchers designed a flexible island-structured electrode featuring primary and secondary root-like protrusions. By laser-cutting PI films with ultraviolet lasers and embedding the resulting structures into stretchable Ecoflex matrices, this design effectively extends the service life of hybrid electronic platforms under diverse mechanical deformations (e.g., stretching, puncturing, twisting) [234]. The structure can endure tensile strains of up to 700% while retaining robust mechanical interlocking and stress dissipation capabilities, thus addressing the interfacial performance limitations between flexible and stretchable substrates in a definitive manner [16].

Currently, the vast majority of microneedles lack stretchability, and it remains challenging to customize the various parameters of microneedle electrodes at the individual device level (e.g., tailored electrode length distributions, detection regions, etc.). This is primarily due to the incompatibility between fabrication processes for rigid microneedle electrodes and stretchable flexible materials, coupled with challenges in material integration and patterning in the construction of three-dimensional microneedle architectures. To address these issues, researchers have developed a hybrid manufacturing strategy that integrates laser micromachining, microfabrication, and transfer printing, yielding a structure composed of polyimide microneedle electrodes and serpentine elastic interconnects. This structure is coated with a gold conductive layer and covalently bonded to a silicone elastomer, thus realizing a highly stretchable (60–90%) microneedle electrode array with exceptional anti-delamination performance [17] (Fig. 8E). However, conventional two-dimensional planar electrodes encapsulated in PI or PDMS substrates exhibit a distinct distance (ranging from several micrometers to hundreds of micrometers) between the recessed electrode sites and the target tissue. This gap can trap air bubbles or induce protein accumulation on the electrode surface, and elevate interfacial impedance. Furthermore, fluctuations in intracranial pressure and brain micromotion may lead to poor contact between the two-dimensional planar electrodes and the dura mater. To address these limitations, researchers designed a 3D soft convex neural microelectrode consisting of a polyimide 3D electrode array (4 channels, scalable on demand) conformally wrapped around the surface of a soft silicone microprotrusion (height: ∼330 μm). This design provides buffered contact capability, reliable mechanical strength, and recording stability that is unaffected by cortical motion. The 3D soft convex neural microelectrode is characterized by a readily scalable fabrication process and robust cyclic compression recovery performance. Following implantation in rats after craniotomy, the electrode captured a sensitive and stable electroencephalogram (EEG) signal during whisker deflection and external pressure application [18].

Integrating liquid metals into elastic substrates to fabricate flexible composite electrodes represents another prevalent strategy. For instance, the poly(α-lipoic acid)-polyvinylpyrrolidone-liquid metal particle composite (PTPL) enables in-situ generation of ionic clusters within the poly(α-lipoic acid) (PTA) network by incorporating polyvinylpyrrolidone (PVP), while simultaneously enhancing interfacial interactions between liquid metal particles (LMPs) and the PTA matrix. This robust interfacial bonding improves electrical conductivity and electrochemical stability, thereby facilitating efficient electrophysiological signal transmission and effectively minimizing signal noise and motion artifacts [19]. Leveraging the thermo-responsive properties of ionic bonds, the PTPL strain sensor exhibits a rapid response and a detection range exceeding 500% strain [235]. Rheological tests demonstrated that with prolonged heating time, the storage modulus (G′) and loss modulus (G″) of the PTP elastomer increased significantly, reflecting enhanced physical crosslinking density of the ionic clusters. Stress-strain curves revealed a concomitant increase in Young's modulus and tensile strength, coupled with a decrease in elongation at break. After 32 h of heating, the PTP elastomer achieved an optimal balance of mechanical properties, with its tensile toughness peaking at 12.12 MJ/m^3^. Steady-state visual evoked potential (SSVEP) experiments verified that PTPL electrodes could accurately identify stimulus frequencies and their corresponding harmonic signals [20]. However, achieving high-precision patterning of liquid metals with line widths below 20 μm on stretchable substrates remains a formidable challenge, primarily due to their inherent fluidity and high surface tension. To address this issue, one strategy is to optimize the stiffness and interfacial properties of rigid islands and flexible regions to realize stretchable electronic devices capable of undergoing large deformations. By in-situ forming rigid islands on flexible substrates—achieving a Young's modulus ratio of nearly four orders of magnitude between the two components and robust interfacial crosslinking—and subsequently printing gallium-indium alloy liquid metals onto the substrate to form hydrogen-bonded interfaces, the resultant devices can maintain stable high conductivity even under tensile strains of up to 600% [236]. An alternative approach involves fabricating liquid-metal-based multilayer solid-liquid electrodes (m-SLEs) via electrohydrodynamic (EHD) printing with confined templates. In this method, liquid metals self-assemble onto high-resolution templates under the selective wetting effect of electrodeposited copper layers. The m-SLEs composed of PDMS/Ag/Cu/EGaIn feature a line width of ≈20 μm and ≈100% stretchability, retaining mechanical stability after ≈10,000 stretch-relaxation cycles and exhibiting recyclability. Notably, the multilayer architecture of these electrodes enables tunable strain–sensing performance [237].

#### 4.1.3 Structural engineering

Beyond material selection, sophisticated structural design represents an effective strategy to endow electrodes with superior flexibility and minimize tissue damage. At the microscale, pore engineering is widely exploited to construct flexible architectures. For instance, nanoporous graphene film technology and associated engineering approaches enable the fabrication of flexible neural interfaces with low impedance and high charge-injection capacity, with favorable biocompatibility validated via chronic implantation [10]. Introducing pore architectures into carbon nanofiber networks yields bamboo-like or golden toad egg-like nanofibers with high flexibility and even transient foldability; the presence of abundant micropores and mesopores further enables the material to withstand repeated folding cycles.

In addition, modulating interlayer interactions represents another viable approach to enhance flexibility. Strengthening interlayer forces confers high tensile strength while retaining favorable mechanical flexibility of the material. Inspired by spider silk, researchers developed water-responsive hypershrinkable polymer films (WRAP films), which undergo rapid and substantial shrinkage upon hydration and transform into soft, stretchable hydrogel films. The shape-adaptive electrode arrays (WRAP electrodes) fabricated on this basis greatly simplify the implantation procedure and have been successfully applied to in vivo neural stimulation and electrophysiological signal recording. Moreover, the dry-state flexibility of WRAP films also facilitates the electronic integration process [225]. A three-layer fibrous mesh microstructure with dual driving forces—pore size gradient and wetting gradient—can strongly adhere to the surfaces of wet tissues with complex curved topographies, forming a conformal interface in intimate contact with the target tissue [21]. At the macroscale, porous architectures also exhibit exceptional flexibility. For example, via serpentine or honeycomb structural designs, the tensile strain range of cellulose-based integrated multilayer microsupercapacitors can be significantly tuned, and the devices maintain stable performance during conformal attachment to curved surfaces and under dynamic deformation conditions [238].

To address the strain-related issues of materials—specifically, the susceptibility of low intrinsic conductivity materials (e.g., graphene, PEDOT:PSS) to resistance fluctuations under strain, as well as the tendency of high-conductivity materials (e.g., silver-based nanomaterials) to undergo oxidative corrosion—researchers have proposed a variety of structural design strategies. One strategy involves fabricating electrodes consisting of two-dimensional (2D) percolation networks (PNs) and protruding PNs. By embedding silver nanowires (AgNWs) or carbon nanotubes into an elastic polymer matrix [22] and controlling their alignment, the electrical conductivity and stability of the electrodes under large strains are significantly enhanced, while the post-implantation inflammatory response is effectively mitigated [23]. Another strategy is to decouple the configuration of bioelectronic materials: tissue interface components are fabricated as brittle thin films with excellent electron transfer properties (e.g., gold, platinum, IrOx), whereas interconnect components are constructed from stretchable, highly conductive materials and seamlessly integrated via an anisotropically conductive film (ACF) adhesive. This design leverages surface-channel cracking in coupled interface components and the anisotropic out-of-plane/in-plane electron conduction mechanism of interconnect components to effectively eliminate the impact of strain on device performance, thereby creating a multifunctional, ultrathin, soft, stretchable, and highly conductive substrate [24]. Even non-ductile and brittle thin films can achieve complete and precise conformal wrapping of delicate surfaces (e.g., microtissues, optical fibers) via an innovative droplet-printing strategy. This technology employs liquid droplets to enable non-destructive transfer of thin films. For instance, in in vivo animal experiments, researchers successfully attached 2-μm-thick silicon films (without any ductility engineering) to the surfaces of mouse nerve and brain tissues via droplet printing, forming conformal neuroelectronic interfaces. These interfaces can modulate in vivo neural activity with infrared light at high spatiotemporal resolution, and their core mechanism lies in effectively preventing in-plane film stretching and reducing stress concentration [221] (Fig. 8F). This research provides a novel paradigm for constructing flexible electronic devices, resolves the issue of stress-induced damage in thin-film adhesion, and offers critical technical support for interdisciplinary fields including flexible electronics and brain-computer interfaces (BCIs).

The miniaturization of material dimensions helps mitigate defect formation and cracking, thereby endowing materials with superior mechanical flexibility. This enables them to withstand minor strains without sustaining damage and integrate with flexible substrates, yielding one-dimensional (1D) electronic devices with excellent bending and stretchability [239]. For instance, the reduced graphene oxide/polyaniline (rGO/PANI) ultrafine electrode (rGPF), with a diameter of approximately 800 nm, exhibits enhanced biocompatibility and minimal physical trauma compared with conventional metal wires and carbon fibers, owing to its low Young's modulus. Despite its submicrometer diameter, rGPF retains sufficient bending rigidity to enable direct, self-supported implantation into the deep brain regions of mice without the need for external auxiliary devices, thus reducing the risk of additional physical injury [25]. Furthermore, researchers have developed a 1D Neuro Worm fiber electrode composed of gold (Au) and styrene–ethylene–butylene–styrene (SEBS), with a diameter of 109 μm. The Neuro Worm electrode has a Young's modulus of approximately 3.1 MPa, which is comparable to that of soft biological tissues. It can withstand tensile strains of up to 94%, accommodate deformations induced by muscle movement, and maintain stable signal acquisition performance. Following 1,000 stretch–relaxation cycles under 30% tensile strain, the Neuro Worm electrode retained a low electrical resistance [26]. In rat models, the device operated stably for over 43 weeks, with minimal impedance fluctuations and consistent signal quality. Histological sections obtained at 54 weeks post-implantation revealed almost no fibroblast encapsulation, and the inflammatory response was significantly attenuated compared with that of conventional rigid electrodes. Endowed with superior mechanical flexibility, biocompatibility, and magnetically actuated propulsion capability, this Neuro Worm electrode overcomes the limitations of traditional implantable electrodes (e.g., immobility and high susceptibility to adverse tissue reactions), offering a viable technical route for next-generation dynamic bioelectronic interfaces (Fig. 8G).

Although flexible electrodes have demonstrated substantial potential in mitigating damage induced by brain micromotion and represent a pivotal direction for the future advancement of neural interface technologies, their inherent limitations cannot be overlooked. First, their extremely low mechanical strength renders ultra-flexible electrodes prone to curling, folding, or fracturing during implantation, increasing procedural difficulty and failure rates. Second, certain ultra-flexible polymeric materials (e.g., some hydrogels) are susceptible to swelling, degradation, or time-dependent deterioration of mechanical properties in the physiological fluid environment, which compromises long-term device stability. Third, while many ultra-flexible materials are deemed biocompatible, their potential long-term toxicity, the biological impact of degradation products, and associated immune responses still necessitate more comprehensive and prolonged in vivo evaluations [240].

The application boundaries and suitable scenarios of the three types of ultra-flexible strategies exhibit clear non-overlapping characteristics. Firstly, the modulus of ultra-flexible hydrogels is fully homologous to that of brain tissue, providing a solution that can thoroughly eliminate mechanical mismatch at its root. It can be combined with anti-inflammatory and anti-fouling functions to achieve multi-functional integration. The core limitations lie in its susceptibility to ion penetration into cerebrospinal fluid, leading to swelling, and in the conductive network's tendency to fracture. It is suitable for cortical ECoG electrodes and peripheral nerve electrodes, but not suitable for deep-brain implantable arrays. Secondly, elastomer processing technology is mature and fully compatible with semiconductor micro- and nano-fabrication processes, exhibiting excellent chemical stability and mature clinical applications. The core limitations are that, even with ultra-thin processing, the modulus remains much higher than that of brain tissue, unable to completely eliminate mechanical mismatch, lacking active regulation capabilities, and still posing a risk of fibrosis. Moreover, it has higher requirements for implantation conditions, making direct insertion difficult and increasing the risk of tissue damage. However, its advantages are also evident, namely higher chip integration, better suitability for clinical-commercial DBS electrodes and high-density cortical recording arrays. Finally, flexible structural design does not alter the material's chemical properties, enabling flexibility and stretchability, and is fully compatible with traditional rigid silicon-based electrodes without affecting their electrochemical performance. The core limitations remain the inability to address the material's inherent modulus mismatch, only buffering macroscopic mechanical deformation, and the inability to eliminate microscale mechanical stimuli. It is suitable for upgrading and optimizing traditional rigid silicon-based arrays and high-density stretchable electrodes.

### 4.2 Self-healing performance

**Fig. 9:**
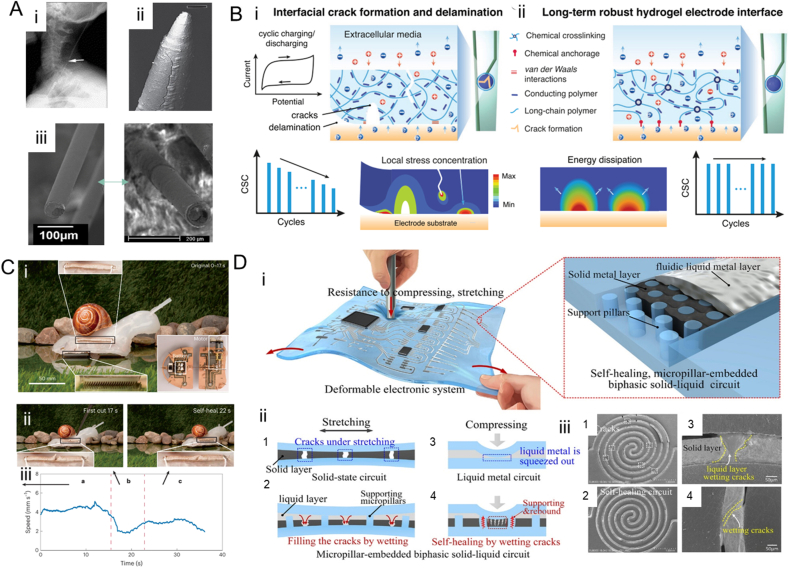
(A) Neural Interface Degradation. i. Wire Failure: Plain radiography showing lead wire breakages in a clinical implant [245]. Copyright 2004, Elsevier. B.V. ii. Coating Fatigue: Side view of a longitudinal crack in the Parylene insulation near surface irregularities after 554 days. Reproduced with permission [246]. Copyright 2014, Frontiers. iii. Corrosion Analysis: SEM and surface relief images showing the calculated corrosion depth of a recording site post-implantation [247]. Copyright 2016, IOP Publishing. (B) Conventional vs. Robust Hydrogel Interfaces. i. Conventional Interface: Schematic showing how cyclic charge injection in standard PEDOT:PSS coatings leads to stress concentration, fatigue cracks, and delamination. ii. Robust Design: A conceptual illustration of PEDOT:Poly(SS-4VP) hydrogel. Chemical anchorage and cross-linking create a tough interpenetrating network that dissipates mechanical energy and prevents interface failure. Reproduced with permission [71]. Copyright 2023, Wiley VCH. (C) Snail-Inspired Self-Healing Robot. i. Design: A fully untethered soft robot using self-healing composites to connect the actuator to the power supply. ii. Recovery: Demonstration of simultaneous mechanical and electrical healing, allowing the robot to resume crawling after damage. iii. Performance: Velocity profile showing the robot's speed before, during, and after the healing process. Reproduced with permission [248]. Copyright 2023, Springer. (D) Biphasic Liquid-Solid Conductors. i. Architecture: A deformable electronic system utilizing a biphasic liquid-solid conductor with embedded micropillars for stretchability. ii. Comparative Mechanics: Structural deformation of solid-state vs. liquid metal vs. biphasic circuits under compression and stretching. iii. Microstructure: SEM comparison of ring circuits; highlights the crack resistance of biphasic liquid-solid metal (2-4) compared to the failure of solid metal (1). Reproduced with permission [249]. Copyright 2025, Wiley VCH.

Secondary surgical trauma must be minimized during the implantation of invasive brain–computer interface (BCI) electrodes. For short-term use electrodes, this goal can be achieved through component degradability: such materials can be controllably degraded into harmless byproducts and subsequently cleared via metabolic pathways in vivo after fulfilling their designed functional lifespan [27,241,242] thereby reducing the risks of infection and secondary tissue damage [243]. However, in long-term implantation scenarios, electronic devices are susceptible to damage induced by excessive stretching, stress fatigue, and stress-initiated microcracks during the continuous movement of organs and joints [8,[244], [245], [246], [247]](Fig. 9A). Furthermore, during prolonged electrical stimulation and signal recording, cyclic capacitive charge injection/ejection can induce localized stress concentrations within the electrode coating, which subsequently lead to the formation of fatigue cracks and interfacial delamination [71] (Fig. 9B). In addition, conductive polymer electrodes remain prone to dedoping or partial degradation in complex physiological microenvironments; this disrupts the conductive percolation network of the material, fragmenting it into disconnected “islands” and severely compromising the long-term operational stability of the electrodes [240]. Although mechanical elasticity can be achieved through various approaches, incorporating self-healing chemical moieties can further enhance the robustness of soft electronic devices, enabling them to recover structural and electrical integrity following mechanical damage [250], and significantly extending the reliability and durability of the electronic system [251,252]. Inspired by the inherent ability of biological tissues to initiate repair via cellular and molecular mechanisms to restore function and morphology after injury [118], self-healing technology has emerged as a core design element for flexible electronics (e.g., electronic skin and sensors). The extensive insights from these fields can provide critical guidance for developing self-healing strategies tailored to implantable BCI electrodes.

Common self-healing mechanisms are categorized into three primary types: (i) Disposable capsule-based mechanism: Mechanical damage ruptures capsules and releases healing agents, triggering physical or chemical repair processes. (ii) Reusable channel-based mechanism: Three-dimensionally interconnected channels continuously supply healing agents, enabling multiple cycles of repair. (iii) Intrinsic reversible bond-guided mechanism: Repair is mediated by dynamic reversible chemical bonds within the material, typically requiring external energy stimuli to initiate [252]. Studies have demonstrated that self-healing materials generally exhibit two key characteristics: (1) a glass transition temperature that governs self-healing behavior and enhances autonomous recovery capability; and (2) tunable crosslinking strength that modulates the material's toughness. Soft gel materials can match the stiffness of native tissues without the need for mechanical adaptation, but they are plagued by dehydration or solvent toxicity issues [253], which may result in performance degradation over time. Therefore, to ensure long-term stable monitoring of biological signals and feedback control, self-healing elastomers are primarily used as substrate materials, encapsulants, or polymer matrices in conductive composites [254].

At the molecular level, self-healing materials achieve damage recovery through a variety of mechanisms, primarily including ionic bonds [62,255], dynamic covalent bonds (e.g., Diels–Alder adducts, disulfide bonds, etc.) [256,257], and dynamic non-covalent bonds (e.g., hydrogen bonds, metal coordination bonds) [258,259]. In addition, the nanoconfinement strategy offers a novel avenue for the design of self-healing materials [260]. For instance, polymerizing monomers in high-concentration solutions and incorporating exfoliated synthetic hectorite nanosheets yields hydrogels with a modulus as high as 50 MPa and a self-healing efficiency of nearly 100% [248] (Fig. 9C). Gel-based self-healing materials are of profound significance for bioelectrodes, with their core advantages manifested in three aspects: (1) Enabling the restoration of structural integrity, functionality, and performance of damaged soft bioelectronic devices; (2) Enhancing mechanical durability via the stress dissipation effect: under mechanical loading, reversible dynamic bonds within the hydrogel act as sacrificial bonds that rupture to dissipate stress and energy, thereby mitigating stress concentration and structural damage; following load removal, these bonds re-form to recover the original structure and mechanical properties, conferring resistance to stress-induced cracking and fatigue [261,262]; (3) Endowing electronic devices with shape adaptability through dynamic bond cleavage and reconfiguration, allowing conformal integration with tissues or organs of varying geometries [250]. Notably, certain self-healing polymer electrodes exhibit autonomous adhesive properties, enabling them to tightly encapsulate nerves without the need for suturing and to alleviate long-term tissue compression via stress-relaxation mechanisms. Six weeks post-implantation, no significant inflammatory or fibrotic responses were detected in the surrounding tissues, demonstrating that self-healing materials hold substantial potential to improve biocompatibility and long-term operational stability [263].

Self-healing substrates and encapsulation materials serve as mechanical supports and protective layers, playing a pivotal role in isolating internal sensitive components from the external physiological environment. However, achieving efficient self-healing at the relatively low temperatures of the human body remains a critical challenge. For instance, styrene–isoprene–styrene/ethylene–vinyl acetate (SIS-EVA) elastomers require heating at 85 °C for 1 h to complete the self-healing process [264]. To address this limitation, researchers have successfully developed an elastomer featuring high toughness, high fracture energy, and a self-healing efficiency of 91% at room temperature, by leveraging the synergistic effects of quadruple hydrogen bonding and slidable crosslinks (polyrotaxanes). Its exceptional performance originates from the dual-crosslinking architecture and the strain-induced crystallization of polyethylene glycol (PEG) within the polyrotaxanes [265]. In addition, poly(glycerol sebacate) (PGS) prepolymers possess a low glass transition temperature and abundant hydroxyl groups; by tailoring the crosslinking density, their modulus can be tuned over a broad range of 0.01–5 MPa, rendering them ideal substrates for room-temperature self-healing elastomers. Based on the Diels–Alder (DA) reaction of furfuryl alcohol-modified PGS, furfuryl alcohol-modified poly(ionic liquid), and bismaleimide, a low-modulus hydrogel-like elastomer was fabricated. This material enables ultrafast self-healing at room temperature via molecular chain entanglement, achieving 98% healing efficiency within 5 s [266]. Collectively, these low-temperature self-healing gels pave the way for in vivo self-repair of brain–computer interface (BCI) electrodes.

The integration of self-healing capabilities with circuitry is critical for brain–computer interface (BCI) electrodes to maintain high conductivity over the long term in the dynamically changing microenvironment of brain tissue and to significantly mitigate signal drift. Inspired by biological skin, a self-healing shape-memory polyurethane acrylate sensor based on disulfide bonds self-repairs upon heating: increased molecular chain mobility facilitates molecular diffusion, entanglement, and chemical bond contact at damaged interfaces [260,267]. The repaired sensor exhibits rapid response and recovery times of 160 ms and 200 ms, respectively [268]. In aqueous environments, ionic gels containing various anions (BF_4_^−^, OTF^−^, TFSI^−^) have been optimized: weak ionic interactions endow polycationic chains and anions with high mobility, enabling the electrode to achieve rapidly self-heals within 10 s [269]. It can restore 96% of its strain limit and 95% of its electrical conductivity within 5 min. When used as soft-circuit interconnects, this ionic gel allows a severed robot to resume crawling [248]. For bladder monitoring devices that undergo volume changes of up to 300%, the self-healing device restores a conductivity of 1000 S/cm within 30 s, and the system rapidly recovers functionality within 5 s after signal interruption caused by damage [270].

For complex curved surfaces such as those of the brain and heart, electronic devices are susceptible to damage induced by friction, torsion, tearing, and compression. To address these challenges, formulating conductive materials into printable inks is an effective strategy for fabricating self-healing ultrathin circuits capable of adapting to complex operational conditions [271]. For instance, when moisture is sprayed onto damaged regions of a blended ink composed of liquid crystalline graphene oxide (LCGO) and silver nanowires (AgNWs), the water-absorbing, swollen LCGO nanosheets undergo partial hydrogen bond dissociation. As these swollen nanosheets come into contact with adjacent LCGO layers, water infiltration is facilitated via mutual diffusion and capillary action; subsequent water evaporation promotes the formation of new hydrogen bonds between neighboring nanosheets, thus enabling circuit reconnection [272]. Furthermore, printable inks have been developed by combining hygroscopic randomly hyperbranched polymers (HRHPs) with solid-liquid biphasic gallium-indium alloys (bGaIn). Leveraging hydrogen bond exchange reactions in HRHPs and the liquid-phase fluidity of bGaIn, the resultant circuits can achieve rapid self-repair and recover a signal-to-noise ratio (SNR) of 44.67 dB. The self-healing and adhesive properties of HRHPs enable the conformal assembly of multiple two-dimensional components onto irregular three-dimensional surfaces (e.g., cubes, saddle-shaped surfaces, hemispheres), demonstrating substantial potential for applications in complex biological environments [273].

To address the issue of compromised circuit integrity induced by tensile deformation, a layered impact-resistant flexible liquid–solid biphasic self-healing circuit architecture offers an effective solution [274]. When liquid-metal-based circuits sustain damage, they can re-establish conductive pathways via self-fusion, ensuring uninterrupted device operation. With well-aligned conductive layers, the electrical self-healing efficiency can even reach 95% [248]. Specifically, this architecture comprises a bottom layer of solid bismuth-indium-tin alloy serving as a compression-resistant substrate, with a top layer of liquid gallium–indium-tin alloy that fills cracks in the solid layer in real time under large deformations. This enables delay-free autonomous restoration of conductivity under tensile and impact loading conditions. In cyclic tests, the biphasic circuit exhibited a mere 0.48% resistance variation after 7,000 compression cycles at 15.9 MPa, and only an approximate 10% resistance increase under 100% tensile strain [249] (Fig. 9D). However, it should be noted that achieving this self-healing functionality requires maintaining a relatively high liquid metal volume fraction (typically ≥50%), which inevitably results in higher liquid metal consumption. This constraint somewhat limits their potential applications. Furthermore, if two adjacent circuit traces are placed in close proximity, the self-healing process may lead to unintended electrical short-circuiting [275].

Self-healing functionality not only helps preserve the stability of electrode electrical performance but also enhances the intrinsic “survivability” of the materials themselves. Instead of directly inhibiting specific inflammatory factors or eliminating bacteria, it acts as a “fail-safe” or “sustaining” mechanism to ensure the integrated material platform maintains stable functionality over the long term in vivo, thereby effectively addressing the inevitable issue of material degradation. Notably, such degradation can precisely serve as a key trigger for exacerbated inflammation (e.g., via the release of hazardous substances) or circuit malfunction. Therefore, self-healing functionality constitutes an indispensable component of long-term stability strategies for implantable electrodes. That said, the current implementation of self-healing functionality in implantable electrodes still faces several critical challenges. For instance, hydrogen bonds may dissociate under prolonged exposure to high-humidity physiological environments, resulting in impedance fluctuations [276]; the intricate design of multi-layer liquid metal architectures is prone to interfacial delamination, which elevates manufacturing costs and complicates maintenance procedures [277]; additionally, the high interfacial volume ratio of nanofillers leads to a drastic stability decline under harsh conditions, potentially inducing localized electrochemical corrosion and accelerating electrode aging [278]. Future research should focus on the development of in-situ, controllable repair technologies characterized by high reliability, efficiency, and precision—such as light- or magnetically triggered self-healing [279] [280,281]—and the construction of artificial intelligence (AI)-enabled self-diagnosis and self-repair systems [282], so as to further advance the practical translation of next-generation intelligent brain–computer interfaces (BCIs).

Capsule-type exogenous self-healing can repair multiple injuries, but its limitations include the neurotoxic risk posed by repair agent leakage and the limited number of repair attempts. Currently, it is only compatible with non-implantable epidermal electrodes and peripheral nerve electrodes. When applying it to brain implantable electrodes, biosecurity issues must be carefully considered. Intrinsic dynamic bond self-healing can achieve cyclic autonomous repair. Compared to the capsule-type self-healing strategy, it is, in principle, more suitable for brain implantable electrodes. However, it also has significant limitations. Most systems require external stimuli such as light and heat for triggering, and precise control cannot be achieved in the enclosed intracranial environment. Dynamic bonds in physiological environments are prone to irreversible breakage, and long-term repair performance continues to decline. Therefore, it is necessary to develop intelligent, mild self-healing stimulation methods that adapt to the intracranial implant environment. In addition, the self-healing function may affect the doping stability of conductive polymers, conflicting with the electrochemical performance of electrodes. Currently, it is only in the laboratory proof-of-concept stage, and intracranial implantation can only be used as an auxiliary strategy for coating modification, making it difficult to serve as the core structural material of electrodes.

Based on the research progress of all strategies in this chapter, ultra-flexible design and self-healing performance are the two core paths to address the long-term implantation failure of neural electrodes. Currently, there are still two deficiencies in this field based on core scientific principles: (i) Existing research generally focuses on optimizing single performance to the extreme, while neglecting the synergistic balance of multiple objectives in intracranial implantation. Long-term intracranial implantation requires the synergistic unification of objectives such as mechanical homogeneity, surgical adaptability, structural stability, and electrochemical compatibility, but most existing research falls into single performance optimization: ultra-flexible hydrogels achieve mechanical homogeneity but are prone to swelling and failure, elastomers meet clinical adaptability but lack autonomous regulation capability, and self-healing systems achieve damage repair but cannot balance the core requirements of intracranial safety and performance compatibility. This is also the core root cause why many technologies in adjacent fields cannot be transformed into a BCI electrode design. (ii) Existing research generally focuses on single-stage performance optimization, while neglecting full-cycle temporal coordination. This is also the core solution direction of the temporal phased design paradigm proposed in this review. Almost all existing research focuses on the independent optimization of single strategies, without integrating the mechanical stability guarantee of this chapter with the acute anti-inflammatory and chronic anti-fouling and antibacterial strategies of the previous two chapters, ignoring the temporal correlation of the full FBR cycle and failing to break the vicious cycle of “micro-motion damage - inflammation - fibrosis - exacerbated mechanical mismatch”. This is also the core scientific root cause of why many electrodes are effective in the short term but fail in the long term.

## 5 Engineering implementation and clinical translation barriers

### 5.1 Engineering requirements

#### 5.1.1 Reliable manufacturing of devices

**Fig. 10:**
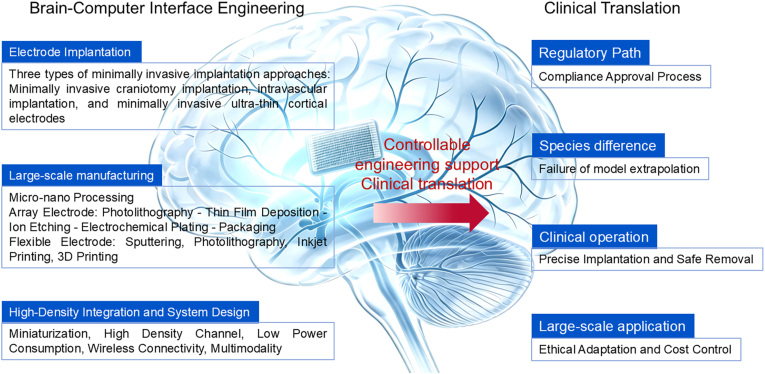
Key points for engineering implementation and barriers to clinical translation of brain-computer interface electrodes.

The engineering implementation of electrodes provides the physical foundation for low-immunogenicity design. Fig. 10 summarizes the key points for engineering implementation and the main barriers to clinical translation discussed in this section. Engineering parameters such as implantation methods, processing techniques, packaging structures, and wireless integration directly determine the degree of tissue trauma, foreign body response, and chronic inflammation after electrode implantation. These parameters are pivotal in bridging material design and clinical biosafety. BCI is an interdisciplinary field involving neurosurgery, materials science, and electronic engineering. Its core consists mainly of precise localization of functional areas, minimally invasive/craniotomy electrode implantation, and the establishment of long-term, stable interfaces. Classification based on surgical pathways is as follows: (i) Classical craniotomy implantation: Implanting electrodes through craniotomy ensures stable signals and precise localization, but it involves significant trauma and a long recovery period. With the advancement of electrode materials, the operation becomes more precise, less invasive, and the brain-computer interaction function improves [283]; (ii) Intravascular implantation (Synchron Stentrode): Implanting electrodes through jugular vein intervention involves minimal trauma and rapid recovery. However, its signal quality is limited, and there is a potential risk of vascular complications [284]; (iii) Ultra-thin cortical surface electrodes (Precision Band): Implanting flexible, integrated high-density electrodes with a thickness of less than 5 μm through a Keyhole small bone window and suspending them in cerebrospinal fluid significantly reduces physical contact, significantly reduces cortical mechanical compression and damage, enhances long-term stability, and achieves high-density, high-throughput neural signal coverage over a large cortical area. However, this method demands extremely high precision in surgical operations and lacks long-term retention stability [285]. Additionally, the depth, angle, and positioning accuracy of implantation directly affect signal quality. With technological advancements, some teams have developed automated implantation systems (Automated Inserters) that can quickly, repeatedly, and precisely complete multi-channel electrode implantation under microscope guidance. The core trends in future implantation surgeries are minimization, wireless integration, full implantation, long-term stability, standardization, and proceduralization of implantation methods. Neuralink's R2 robot achieves automated surgery with 1.5 s per electrode and less than 2 h for the entire procedure, without the need to incise the dura mater, significantly reducing surgical risks. Meanwhile, a dedicated ASIC chip integrates multi-channel acquisition, real-time processing, and wireless transmission. Minimally invasive implantation can reduce mechanical damage to brain tissue and acute immune activation from the source, serving as the primary engineering means to reduce electrode immunogenicity and postoperative glial scar proliferation.

In terms of hardware, ultra-high channel counts and ultra-flexible electrodes are the main development focuses. For example, Precision Neuroscience, a US company, has developed the Layer 7 surface-type thin-film array, which supports temporary mapping of more than 1024 channels and has currently obtained approval for temporary implantation testing. Blackrock Neurotech's Neuralace flexible grid array aims to achieve more than 10,000 channels. The Neuralink team continues to iterate on thread design and channel count improvement, and is pushing the Blindsight vision restoration chip into the preparation phase for human testing. The preparation of microarray electrodes involves a series of processes, including photolithography, thin-film deposition, etching, electrodeposition, and encapsulation. Silicon-based electrodes often use MEMS technology. On a silicon wafer, electrode patterns are formed by photolithography, followed by metal sputtering and deep reactive-ion etching to produce a three-dimensional needle array.

ECoG electrode arrays can be divided into passive and active types. The passive array is composed solely of electrodes and insulating layers, has a simple design, high spatial resolution, and can directly measure the film potential. However, due to the physical size of interconnects, it is necessary to balance the electrode density, the number of electrodes, and the coverage area. In contrast, the active array improves signal fidelity and spatial resolution through a multiplexing topology and active devices, while significantly reducing the number of interconnects. The array can be expanded without sacrificing electrode density and coverage area. However, this poses a challenge to the material performance, requiring the material system to have excellent transient response, fast switching, scalability, and manufacturing compatibility. For example, the active array based on the MoS_2_ semiconductor realizes multiplexing and sensing functions through a highly scalable, monolithic manufacturing process without transfer, and a dual-transistor multiplexing-source-following architecture in each pixel. To achieve large-scale integration, the team directly grew wafer-level three-layer low-temperature MoS_2_ on the PI substrate and manufactured eight 10 × 10 active arrays, which were further expanded to a high-density 2,500-channel, 51-sites/mm^2^ matrix [286]. In vivo, the multi-channel array synchronously recorded brain signals with high spatial resolution, achieving 16 sites mm^−2^ in AEP imaging and 51 sites mm^−2^ in MUA recording. The size of the traditional ECoG electrode is millimeter scale, which does not match the size of the cortical column with the size of tens to hundreds of micrometers, and it is difficult to meet the requirements of high resolution; High density micro cortical electroencephalogram (lECoG) array is faced with the problems of complex passive electrode wiring, complex active array manufacturing process (such as monocrystalline silicon, graphene based TFT), high cost and difficult to scale. Neurocam, a 4096-channel flexible lECoG device based on an Ln-IZO TFT array, breaks the wiring bottleneck of high-density arrays through time-division multiplexing, enables large-scale, low-cost production of the device through an industrial manufacturing process, and can achieve high-yield manufacturing of 20 cm × 20 cm large substrates [287].

However, traditional microelectrode arrays are typically planar, making it difficult for them to effectively interact with neurons distributed in three dimensions. Therefore, while existing silicon-based microelectronic technologies can record and modulate neural activity with high spatiotemporal resolution, their planar form presents significant limitations when targeting three-dimensional neural structures. To address this limitation 3D printing technology offers significant advantages in microelectrode manufacturing efficiency, including direct fabrication without post-processing, high conductivity and biocompatibility, and flexibility in microstructural design. A new method has been proposed that combines high-resolution 3D printing (via two-photon polymerization, 2 PP) with scalable microfabrication technology to directly fabricate tissue-penetrating 3D microelectrodes on planar microelectronic devices. This method allows researchers to customize the shape, height, and position of the electrodes as needed, enabling precise localization of neuronal populations distributed in three-dimensional space. The research team successfully customized the microelectrode array using this method to effectively interact with retinal ganglion cells (RGCs) in the retina, ensuring that the height and shape of the microelectrodes precisely match the distribution of target neurons [288].

Hydrogel electrodes generally adopt more traditional casting and coating methods or advanced manufacturing processes such as photolithography and 3D printing [289]. Composite flexible electrodes often incorporate polymer substrates (such as PI or Parylene-C), with lower processing temperatures to ensure material flexibility. Conductive patterns are formed by sputtering thin metal layers (such as Au or Pt), followed by an insulating protective layer, to achieve a multi-layer wiring structure. To reduce costs and improve consistency, some studies have adopted roll-to-roll and inkjet printing technologies, offering opportunities for the mass production and single-use of electrodes in the future. These processes not only determine the geometric accuracy of the electrodes but also directly affect their electrical performance and long-term reliability. The structural design of flexible electrodes is also becoming increasingly diverse, such as multi-layer sandwich-style conductive structures, grid-type arrays, and stretchable corrugated structures, to balance signal transmission and structural stability. However, the implantation of such electrodes is more complex, and they are prone to bending or curling as they enter brain tissue, requiring the use of soluble supports or rigid pins. In addition, the encapsulation and connection technology of flexible substrates is also an engineering challenge, requiring a balance between flexibility and sealing. Moreover, most current microneedles lack stretchability, making it difficult to customize parameters such as electrode length distribution and detection area at the individual device level. This is mainly due to the incompatibility between the manufacturing process for rigid microneedle electrodes and stretchable, flexible materials, as well as the challenges of integrating and patterning materials within the three-dimensional microneedle structure. It is necessary to integrate various manufacturing processes, such as laser micromachining, replication molding, micromachining, and transfer printing, to manufacture composite electrodes while simultaneously stretching the microneedle array and the substrate [17]. Furthermore, the surface regularity and structural uniformity of micro-nano processing directly affect the biological compatibility of the electrode–brain tissue interface, which is a basic condition for maintaining stable interfaces after implantation and for reducing local tissue stress.

With the increase in channel count and the compression of implant volume, developing a fully integrated application-specific integrated circuit (ASIC) for brain–computer interfaces, integrating acquisition, transmission, stimulation, and power management onto a single chip as much as possible, is the only way to achieve device miniaturization and ultra-low power consumption. To address the bottleneck of massive data transmission caused by high channel counts, ultra-wideband wireless transmission chips based on advanced technology nodes are also being planned. Compared to traditional low-power Bluetooth, ultra-wideband technology can provide tens of times the transmission bandwidth with a reasonable increase in power consumption, providing physical support for the wireless transmission of raw data from hundreds or even thousands of channels in the future.

However, in the brain-computer interface industry chain, the academic community currently focuses on the innovation of flexible electrode materials, neural decoding algorithms, and large model applications, yet there is little systematic analysis of the underlying engineering logic of brain-computer chips. The chip signal acquisition architecture mainly comprises two mainstream technical approaches, each suited to different physiological signal extraction needs. The synchronous acquisition architecture, as implemented by TI, integrates multiple on-chip ADCs in parallel, with each channel sampling strictly synchronously in the same clock cycle, providing high precision and excellent channel phase consistency. It is suitable for scalp and cortical EEG acquisition and can meet the stringent requirements for absolute timing synchronization in large-scale brain region network coordination and neural circuit research. The asynchronous acquisition architecture, typified by the Intan solution, employs a multiplexer paired with a single high-speed ADC to perform high-speed polling sampling on multiple electrode channels. It significantly reduces chip area at the expense of absolute synchronization, supports scalability to 64, 128, or more channels, and is suitable for single-neuron action potential acquisition using high-density microneedle electrode arrays. Action potentials have higher amplitude and higher tolerance for sampling accuracy, but require a stringent channel density. Therefore, the performance evaluation of the acquisition architecture needs to closely consider the adaptability of electrode morphology to neural targets. The essence of architectural selection lies in a multidimensional trade-off among accuracy, channel density, sampling rate, and power consumption.

The available space inside the brain is extremely limited, requiring the BCI chip and peripheral circuits to be extremely miniaturized. At the same time, there are rigid constraints on the temperature rise of implanted devices. Human brain tissue is highly sensitive to temperature, and a temperature rise exceeding the tolerance threshold can cause irreversible neuronal damage. This requires the chip to eliminate redundant functions and reduce leakage current and dynamic power consumption to physical limits. Contrary to common sense, the lifespan of a BCI does not lie in the chip. The core bottleneck that truly limits its clinical service life lies in the electrode materials and overall packaging. The brain is not a static fluid environment, but a complex microenvironment with continuous immune rejection and dynamic chemical changes. According to academic consensus, when electrodes come into direct contact with neural tissue, long-term inflammatory responses can induce glial scar formation, leading to a sharp increase in electrode impedance and continuous attenuation of neural signals. At the same time, body fluids can cause electrochemical corrosion of precious metals and conductive polymers on electrode surfaces. The overall packaging must provide electrical insulation and physical isolation between the silicon-based chip and the body fluid environment, while also allowing the penetration of a large number of micro-wires through the packaging for electrode interconnection. An increase in the number of channels leads to a geometric increase in pin density, making the high-density feedthrough process significantly more difficult. In addition, microscale sealing failures and moisture leakage can cause short circuits and damage to integrated circuits, and packaging yield directly determines the feasibility of industrialization. Currently, common insulating materials for BCI chips include polyimide (PI), Parylene-C, silicon dioxide, and silicon nitride, with waterproofing, impermeability, and structural stability as the core goals. Flexible electrodes often use multi-layer laminated packaging to balance flexibility and ion-barrier performance. Traditional dielectric elastomers can be used as soft packaging materials for bioelectronics, but because the molecular permeability of soft polymers is typically several orders of magnitude higher than that of plastics and inorganic materials, ions in physiological solutions will gradually permeate into the elastomer, affecting its long-term packaging performance [290]. With the development of miniaturization and multi-channelization of devices, packaging is evolving towards lightweight, high-integration, and integrated integration of wires, insulation, amplification circuits, and connection interfaces on a single substrate.

Traditional implantable BCI electrodes often rely on percutaneous leads or large external induction coils, which are prone to causing infections, chronic tissue inflammation, and mechanical traction injuries. Additionally, they significantly increase the risks of surgical trauma and device failure, severely limiting long-term implant stability. To address this, researchers have developed various innovative wireless power and signal transmission solutions tailored for BCI electrodes, including far-field radio frequency (RF) radiation, near-field induction coupling, mid-field electromagnetic induction combining induction and radiation modes, ultrasonic waves, and light energy. These provide core technical support for achieving fully implanted, miniaturized, low-trauma, and wireless electrode operation. These approaches are summarized below.

(i) Electromagnetic RF transmission type: The fully wireless architecture employs inductive coupling for wireless power supply and utilizes ultra-wideband (UWB) technology for data transmission, completely eliminating the need for percutaneous cables and subsequent data transmission. It allows for the integration of microelectrodes onto ultra-thin silicon films, with the system chip and flexible packaging fused, avoiding the traditional “electrode + backend processing” separation architecture and achieving a high-bandwidth, fully wireless power supply and data transmission architecture [285]. (ii) Optical signal regulation type: The optical brain-computer interface is a novel neural interaction technology that uses “light” as the information carrier to read, decode, and regulate neural signals. Compared to traditional electrical brain-computer interfaces, optical brain-computer interfaces exhibit unique advantages in spatial resolution, cell specificity, and multi-parameter synchronous monitoring. The fully implantable wireless transcranial optogenetic stimulation system, centered on a programmable micro-inorganic LED (μ-ILED) array, can generate multi-regional and multi-temporal optical stimulation patterns in real time within the cortical layers of a living mouse brain, successfully inducing learnable and distinguishable artificial perception. The system integrates a wireless control module with a flexible optogenetic display panel (FOD) and employs flexible materials for integrated packaging. Its wireless energy is provided by a 13.56 MHz external antenna, and the internal module powers the μ-ILEDs via magnetic coupling to drive them to emit light. Researchers can precisely regulate the on-off timing and emission intensity of individual μ-ILEDs through MATLAB real-time programming, encoding optical stimulation into standardized digital brain signals, thereby achieving precise optical regulation of neural activity. Compared to existing intracranial electrical stimulation techniques, it can cover a larger cortical area, is expected to achieve more precise neural population regulation, possesses high stability and scalability, and can be adapted to various experimental models ranging from rodents to non-human primates. Future research focuses include: improving the spatial resolution of optical stimulation by reducing μ-ILED size and increasing array density to achieve more precise regulation of neuronal clusters; and integrating real-time neural recording modules to construct a “stimulation-recording” closed-loop system for adaptive regulation of neural activity [291]. (iii) Magnetic coupling drive type: Magnetoelectric (ME) materials serve as a wireless data and energy transmission technology. Compared to other wireless power supply technologies for bioelectronic implants, ME technology has advantages such as high-power density, strong tolerance to offset, and the ability to operate in deep tissues. The magnetic field can be efficiently converted into biologically relevant signals such as electricity, heat, or mechanical signals, enabling remote energy transfer and neural activity detection [292]. Using magnetoelectric (ME) materials to achieve wireless power supply and data transmission: The ME bilayer film consists of a magnetostrictive layer (M) for efficient energy harvesting; simultaneously, digital signals are transmitted by modulating the magnetic field frequency, with frequency changes being converted into changes in received voltage amplitude, which are decoded into stimulus parameter commands. The entire system comprises an external magnetic field emitter, an ME energy and data acquisition module, a customized ASIC processing circuit, and stimulation electrodes [293]. The challenges that need to be addressed for magnetically controlled electrodes include signal cubic attenuation due to implantation depth, local overheating from magnetocaloric runaway, and strong electromagnetic interference (EMI) generated by the device itself. This “noise” can severely contaminate or even completely drown out weak neural electrical signals. (iv) Ultrasound-mediated type: It replaces traditional invasive implanted electrodes with focused ultrasound to achieve completely non-invasive bidirectional brain signal interaction. Ultrasound can safely penetrate the skull and precisely focus on millimeter-scale neural targets deep within the brain, enabling both the reading of neural activity signals and the precise regulation of neuronal firing. This fundamentally avoids the core pain points of invasive solutions, such as surgical trauma, infection, and rejection. Ultimately, to achieve high-throughput, high-spatial-precision bidirectional signal interaction across the entire brain, deep fusion and bidirectional enhancement of human brain and AI capabilities are achieved, expanding to full-scenario applications such as sensation and motor control. The advantage of the electrical route lies in “reading”: invasive electrodes directly record neuronal firing with time resolution down to microseconds, which ultrasound currently cannot match. However, ultrasound excels in “writing” and “coverage”, meaning it can reach deep within the entire brain and switch targets arbitrarily. Moreover, ultrasound is a mechanical wave, and its emission does not cause severe electromagnetic interference to simultaneously recorded neural electrical signals, which also facilitates the development of multimodal closed-loop ultrasound regulation. However, the attenuation of ultrasound by bones is approximately 1.3 dB/cm·MHz, and high-frequency ultrasound can attenuate by up to 70%, which is a physical bottleneck limiting reading accuracy. Currently, demonstrations rely heavily on cranial windows or semi-invasive acoustic windows, and true non-invasive reading across the entire brain through intact cranial bones has not yet been achieved [294].

Ultimately, these wirelessly transmitted brain-computer interface systems still face a common issue: after miniaturization, or even microminiaturization, the high integration and energy-consumption management demands require the development of dedicated integrated chips.

#### 5.1.2 Multimodal BCI

Multimodal BCI is a brain–computer interaction system that integrates multiple sources of neural or physiological signals. By synchronously acquiring and intelligently fusing diverse types of brain activity data [295], combined with advanced algorithms that decode brain intentions, it enables more efficient and robust direct information exchange between the human brain and external devices. It differs from traditional single-modality BCI, with the core in the collaborative fusion of heterogeneous multi-source data, compensating for the limitations of single signals and significantly enhancing system performance. We categorize multimodal BCI into two main types based on its working mode: (i) Multimodal Signal Acquisition Paradigm: Breaking through the limitations of traditional single-mode brain neural electrical signal acquisition, it relies on electrode arrays to simultaneously obtain brain electrical signals and multiple types of brain physiological and functional signals, such as blood oxygenation, fluorescence, and electromyography, enriching the decoding dimensions of neural information. (ii) Multimodal Neural Stimulation Paradigm: Using electrodes and supporting functional devices as carriers, it integrates multiple physical stimulation modalities such as electrical, optical, and magnetic, achieving multi-dimensional, precise regulation and functional intervention of brain nerves. Traditional neural interface technologies have always faced numerous bottlenecks. Currently, widely used flat-ended optical fibers can only interact with nerve tissue at the distal tip, limiting spatially precise effects across brain layers. Rigid neural probes, due to their mechanical mismatch with soft brain tissue, are prone to causing tissue damage and strong inflammatory reactions in chronic implantation experiments. Meanwhile, traditional methods require separate devices for optogenetics, drug infusion, and electrophysiological recording, making experimental setups complex and difficult to conduct real-time multimodal brain circuit research. Therefore, there is a need to develop novel brain-computer interface solutions that are compatible with multimodal working models.

A research team has designed an innovative microfluidic axial electrode (mAxialtrode). Its core is a thermally drawn step-index polymer fiber, with polycarbonate (PC) as the core and polymethyl methacrylate (PMMA) as the cladding. The team prepared macroscopic preforms using the rod-and-tube method and drilled eight blind holes at the lower end of the PMMA tube to maintain stable pressure within the channel during thermal drawing. In a single batch, hundreds of meters of flexible polymer fibers with diameters ranging from 420 to 440 μm can be successfully prepared. The fiber core is a 200 μm optical waveguide, surrounded by eight evenly distributed 20 μm microfluidic channels, enabling simultaneous optical transmission, electrode integration, and drug delivery [296]. Near-infrared spectroscopy (NIRS) can be used to supplement EEG in signal acquisition, as NIRS has good spatial resolution but low temporal resolution. The synchronous recording of the two can complement each other and enhance recognition performance [297]. EEG records neuro electrophysiological signals (power spectral density, PSD; event-related desynchronization, ERD) at 1 kHz using 32-channel electrodes, while NIRS monitors hemodynamic indicators, such as oxyhemoglobin, in the brain in real time at 11 Hz through 45 optical channels. The channels cover key brain regions, including the bilateral prefrontal cortex, motor cortex, and occipital lobe, enabling synchronous acquisition of neuroelectrophysiological and hemodynamic signals. By quantifying patients' motor intention (MI), the accuracy of diagnosing consciousness disorders has been significantly improved, providing an objective solution to the problem of high misdiagnosis rates in traditional assessment methods [298]. In addition, temperature sensors can be integrated to continuously monitor electrical, optical, and thermal signals in the brain in real time. By utilizing the photoelectric effect of silicon-based transistors and the functional reuse of active components, the system integrates a temperature sensor for thermal sensing (accuracy 0.1 °C) and precise photodynamic measurement (0.57 nA/(mW/cm^2^)) of optogenetic signals through the capacitive coupling mechanism (signal-to-noise ratio ∼40 dB) for weak brain signals. The system maintains the mechanical flexibility necessary for long-term, stable contact with neural tissue, and its multifunctional operating modes do not interfere with one another. Through continuous multimodal neurodynamic data acquisition, potential diagnostic and therapeutic approaches can be provided to improve the quality of life of patients with chronic neurological diseases, especially epilepsy and brain tumors [299].

The development of invasive multimodal brain–computer interface electrodes is constrained by three major underlying coupling constraints: (i) Biological constraints: there is an inherent contradiction between the requirements for long-term implantation biocompatibility and foreign body reaction suppression, and the tissue trauma and increased surface area of foreign bodies caused by multimodal integration. (ii) Physical constraints: the strict limitations on intracranial space and brain tissue thermal safety conflict with the increased power consumption density and structural complexity resulting from multi-unit integration. (iii) Electrical constraints: the demand for low noise and high bandwidth in multimodal signal acquisition is difficult to reconcile with the channel crosstalk and increased site impedance caused by high-density integration. Under these constraints, the core bottlenecks of multimodal electrodes become prominent: the difficulty of integrating multifunctional units into a single unit, and the poor compatibility of cross-modal material interfaces, which easily leads to isomorphic signal timing/spatial misalignment and uneven signal-to-noise ratio. At the same time, high-density integration demands extremely high adaptability to the microstructure and physicochemical stability of materials, and conventional electrodes cannot achieve electrical–optical-biochemical multimodal collaborative sensing and regulation. Therefore, targeted research at the material level is urgently needed, with a focus on developing flexible composite substrates compatible with multifunctional integration, low-impedance stable conductive interfaces, electrical–optical–biochemical coupling sensing materials, anti-inflammatory and anti-scarring modifying coatings, as well as corrosion-resistant self-healing encapsulation and miniaturized energy-supplying materials, to fundamentally solve the problems of multimodal integration compatibility, signal stability, and long-term service. The material platform design concept proposed in this paper, which matches the electrode function with the implantation sequence, provides a core theoretical basis for the rational design of multimodal electrodes.

### 5.2 Core barriers of clinical transformation

At present, global invasive BCI electrode implantation is still in the early stages of small-scale testing and human data collection. The full lifecycle safety of long-term implantation has not been fully verified, which is the primary premise and regulatory core access threshold for its clinical transformation. There are fewer than 100 volunteers worldwide who can implant long-lasting nerve probes, and most studies are still short-term trials. Although a number of devices have been approved for experimental studies, only a few invasive BCI products worldwide have been approved by the FDA and CE for limited indications, and the industry lacks unified clinical evaluation, performance verification, and adverse event control standards. For example, the Reconate system from Motif Neurotech in the United States has been approved by the FDA. It is only allowed to carry out clinical studies to collect safety and preliminary effectiveness data, and is not officially approved for marketing. Problems such as a lack of R&D compliance design, unclear clinical endpoints, and vague indications have become the core top-level barriers to industry implementation. China issued the Chinese expert consensus on the management of the clinical application path of implantable BCIs, which standardized clinical research in areas such as ethical review, informed consent, and safety monitoring, and promoted the orderly development of technology.

The existing laboratory validation is mostly based on short-term animal experimental data, while the clinical application requires the device to have a long-term stable working ability of more than years. There is no mature standardized prevention and control scheme for various complications, nerve tissue injury caused by device performance degradation, chronic glial scar hyperplasia, and other clinical adverse events. Moreover, there are species differences between animal models and human brain, and there are essential differences in the immune microenvironment, nerve cell composition, and physiological response mode of brain tissues of different species. For example, there are orders of magnitude differences in the proportion of immune cells and inflammatory response mode between rodent brain tissues and human brain. Therefore, the neural response characteristics of non-human primate models and the foreign body reaction law after long-term implantation cannot be completely linearly extrapolated to human clinical application scenarios. At present, the in vivo performance and safety of invasive BCI are evaluated by selecting materials and coatings in rodents, then verifying the implantation process and long-term stability in pigs/sheep. Previous studies have shown that dural opening in primates may lead to brain tissue displacement due to loss of cerebrospinal fluid (CSF), air inflow, and reduced intracranial pressure. Compared with rodent brains, the large deformation and movement of primate brains pose additional challenges for building a reliable electrode–tissue interface. The electrode design should have high extensibility and a floating design, which can effectively accommodate the large dynamic movements and deformations of the primate brain [300]. And it is still necessary to complete the evaluation of behavioral performance and decoding accuracy in non-human primates [301], because their neural discharge patterns are closest to those of humans and can better simulate the feasibility and neural responses to electrode implantation [300,302]. In large-animal and human brains, chronic material aging driven by mechanical mismatch exerts the strongest rate-limiting effect on long-term stability, because persistent micro-trauma from cardiac and respiratory cycles continuously re-injures tissue and fatigues electrodes. Acute immunomodulation or anti-fouling coatings alone are insufficient without a mechanically compliant interface. To disentangle contributions: (1) orthogonal comparisons — implant soft vs. rigid probes with identical surface chemistry to isolate mechanical effects; test rigid probes with vs. without anti-fouling coating to isolate biofouling. (2) Staggered interventions — use coatings that deliver anti-inflammatory agents only in the first month vs. long-term active coatings, adding a temporal dimension. Such experiments would transform descriptive understanding of the foreign body response into a predictive framework for clinical translation.

Traditional high-bandwidth BCI relies on invasive surgery or brain-penetrating electrodes, resulting in tissue damage, aggravated by the increase in the number of electrodes, difficult electrode removal, and poor long-term stability (e.g., the neural decoding performance of penetrating electrodes decays over time). In addition, the existing clinical implants are limited to a small number of highly customized patients, and it is difficult to expand to a large population, because the operation time, risk, and the number of channels have increased disproportionately. The ideal BCI system needs to achieve minimally invasive, reversible implantation and support high-density data acquisition across multiple brain regions to accommodate the leap in channel count from thousands to tens of thousands in the future [283].

However, the risk of removing the implanted electrode and the clinical operation specification are blank. In the event of adverse events, the investigator shall provide timely and adequate treatment to the subjects. The investigator shall report to the institutional management department and the ethics committee within 24 h after learning of the serious adverse event. When it is found that the risk exceeds the possible benefits and the study needs to be suspended or terminated, it is necessary to ensure that the subjects receive appropriate treatment and follow-up. However, adhesion and fibrosis between the device and brain tissue after long-term implantation can lead to severe clinical complications, such as nerve tissue tears and intracranial hemorrhage during electrode removal. At present, the industry has not yet formed a unified clinical operation specification and risk grading control standard for electrode removal, which has become a key bottleneck restricting the development of invasive BCI from clinical trials to large-scale clinical applications. For short-term implantation needs, wireless transmission and biodegradability are excellent solutions to address long-term implantation safety. Uncontrolled degradation may lead to premature circuit failure. For the removal of long-term implanted electrodes, it is necessary to fully consider the selection of implantation cycle in the design of electrodes to build an electrode material platform, which is also the problem this review attempts to solve, such as antifouling, lubrication, and the introduction of inert surfaces, rather than leaving the pressure to patients and clinicians. In addition, the optimal design of electrode shape and size can reduce immune activation and improve cell compatibility; environmentally responsive dynamic materials can enhance the potential for reversible implantation, improve implant safety, and reduce surgical complexity. Relevant design strategies can support minimally invasive, reversible implantation and clinically safe removal [303]. In addition, the sterilization compatibility of implantable brain-computer interface electrodes has not been systematically evaluated. Common ethylene oxide or gamma irradiation may cause performance degradation of the flexible polymer substrate, the conductive polymer coating, and the micro-nano structure, which is a key process to be solved in the early stage of clinical transformation.

On the whole, the imperfect ethical system and high cost together limit the clinical implementation and promotion of the brain-computer interface. As a minimally invasive and invasive neuromedical technology, its clinical use must adhere to the core requirements of minimal injury and supplement ethical rules, such as long-term implant safety supervision, nerve privacy protection, and the definition of the scope of technology application as soon as possible. Meanwhile, the overall cost of device research and development, surgical implantation, and postoperative rehabilitation is high, and there is a lack of medical insurance support and cost-reduction approaches for large-scale production, which make it difficult to popularize the technology [304].

## 6 Outlook and challenges

This review identifies three interconnected core challenges in the design of implanted electrodes regarding biocompatibility and long-term stability, which represent distinct stages within the dynamic evolutionary process of the electrode-induced foreign body response (FBR). Specifically: (1) Acute phase immune response: Short-term FBRs and acute immune responses post-implantation, including inflammatory responses triggered by protein adsorption and immune activation resulting from blood-brain barrier disruption; (2) Long-term interface degradation and biological pollution: Long-term issues such as non-specific protein adsorption, cell adhesion, chronic inflammation, electrode-tissue interface degradation, and signal attenuation induced by glial scar encapsulation, as well as potential risks of bacterial colonization and infection; (3) Material aging and mechanical mismatch: Long-term in vivo aging and corrosion of the electrode material itself, along with chronic fretting damage, persistent local inflammatory responses, and exacerbated fibrotic encapsulation caused by the mechanical modulus mismatch at the electrode-tissue interface. We contend that addressing these three challenges is paramount for the stable and effective application of BCI electrodes.

These factors are not isolated; they interact to form a complex positive feedback cycle that determines the long-term success or failure of BCI function. The intense inflammatory response during the acute phase lays the foundation for subsequent biofouling and chronic inflammation. The continuous adhesion of biofouling components, coupled with the inflammatory microenvironment, significantly promotes biofilm formation, thereby increasing electrode impedance and impairing signal transmission efficiency. Long-term mechanical stress misalignment, material aging, and the chronic fretting damage induced thereby further exacerbate the development of chronic inflammation and glial scarring. Therefore, a single-component material is insufficient to meet the diverse performance requirements of neural electrodes. By applying composite materials or surface-modifying base electrodes, it is anticipated that greater performance benefits can be achieved, facilitating the development of ideal neural electrodes and the realization of seamless brain-computer interfaces. Nevertheless, it should be acknowledged that while multifunctional coupling can endow electrodes with more integrated performance, it may also introduce functional conflicts and non-linear performance gains. For instance, integrating drug delivery and signal acquisition functions at the same site enhances overall integration but compromises the accuracy of each individual function [305].

Similarly, introducing hydrophilic polymer chains to achieve anti-biofouling performance may lead to excessive polymer coverage of active sites, creating a direct conflict between interface stability and electrochemical activity [71]. Likewise, microphase separation between self-healing components and conductive phases generates an interfacial energy barrier, resulting in lower-than-expected conductivity after self-repair. The core contradiction lies in the topological incompatibility between the dynamic bonding network and the continuous conductive pathway [250]. Therefore, the rational design of neural electrodes should not pursue perfection in all aspects, but rather strategically weigh and optimize key functions based on the target implantation cycle and application scenarios.

The way to resolve these conflicts is not to find a single perfect material, but to develop a design strategy that is explicitly tailored to the implementation context. This means setting clear priorities based on two key factors of the device's expected service life and the anatomical target region. For an electrode intended to function for only a few weeks in the cerebral cortex, the primary goals are to suppress the acute inflammatory response and to prevent biofouling from degrading signal quality. In this scenario, a modest reduction in electrical conductivity may be acceptable if it results in a more biocompatible, fouling-resistant interface. Conversely, for a chronic implant designed to record from deep brain structures for years, the long-term mechanical stability becomes the absolute priority. Here, a material's ability to match the brain's softness, resist fatigue, and even self-heal micro-damage is more critical than achieving the lowest possible initial impedance. Computational models can play a decisive role in navigating these trade-offs before conducting costly, time-consuming in vivo experiments. For instance, finite element analysis can simulate how an electrode's stiffness and shape will affect surrounding tissue stress and predict the likely extent of chronic glial scarring. Molecular dynamics simulations can help visualize exactly when a highly hydrated anti-fouling coating begins to impede the charge-transfer process required for signal recording. By integrating these physics-based models with machine learning, it becomes possible to efficiently screen large numbers of material combinations and structural designs. For example, algorithms could be used to identify coating compositions that minimize long-term impedance increase while maintaining protein adsorption below a defined threshold. Ultimately, the goal is to create neural interfaces that are not just generally biocompatible but are precisely engineered to meet the specific demands of their intended use in the body.

Overall, the essence of achieving long-term integration between implantable electronic devices and the nervous system is to construct a bio-functional interface capable of dynamically responding to changes in the brain microenvironment. This demands that we move beyond the traditional “biological inertia” mindset and instead design a multifunctional material integration platform endowed with metabolic adaptability, immune homeostasis-maintaining capacity, and neural affinity. Its value resides not only in supporting high-fidelity signal acquisition but also in establishing a sustainable in vivo survival foundation for BCI systems. The temporal stage-specific design paradigm proposed in this review breaks through the limitations of conventional single-stage intervention and multifunctional superposition strategies, and provides a systematic, full-lifecycle design logic for the development of long-term stable neural interface materials. In the future, to realize an ideal neural interface characterized by high performance, long service life, and multifunctional integration, it is imperative to develop a highly biocompatible material integration platform based on an in-depth understanding and collaborative optimization of the implant-associated FBR issue. Ultimately, these more complex functional requirements for neural information processing and modulation should be organically integrated with this material platform. This will pose unprecedented challenges to the in-depth collaboration and theoretical model innovation across multiple disciplines, including materials science, bioengineering, microelectronics, neuroscience, and computational science.

## Ethics approval and consent to participate

As a review article, this manuscript synthesizes and analyzes previously published literature and does not involve any new studies with human participants or animals. Therefore, ethical approval and consent to participate were not required.

## CRediT authorship contribution statement

**Wei Yao:** Conceptualization, Data curation, Investigation, Methodology, Project administration, Software, Supervision, Visualization, Writing – original draft. **Yueyue Li:** Methodology, Visualization, Writing – original draft. **Yuanyuan Meng:** Validation. **Xiaoling Pan:** Investigation. **Zhenyu Sun:** Methodology. **Yuan Gao:** Methodology. **Xiaodong Zhu:** Conceptualization, Funding acquisition, Project administration, Resources, Supervision, Writing – original draft.

## Declaration of competing interest

The authors declare that they have no known competing financial interests or personal relationships that could have appeared to influence the work reported in this paper.

## Data Availability

No data was used for the research described in the article.
